# Time Is of the Essence—Early Activation of the Mevalonate Pathway in Apple Challenged With Gray Mold Correlates With Reduced Susceptibility During Postharvest Storage

**DOI:** 10.3389/fmicb.2022.797234

**Published:** 2022-05-12

**Authors:** Matthias Naets, Wendy Van Hemelrijck, Willem Gruyters, Pieter Verboven, Bart Nicolaï, Wannes Keulemans, Barbara De Coninck, Annemie H. Geeraerd

**Affiliations:** ^1^Division of MeBioS, Department of Biosystems (BIOSYST), KU Leuven, Leuven, Belgium; ^2^Research Station for Fruit Cultivation, Department of Mycology, Sint-Truiden, Belgium; ^3^Flanders Centre of Postharvest Technology (VCBT), Leuven, Belgium; ^4^Division of Crop Biotechnics, Department of Biosystems (BIOSYST), KU Leuven, Leuven, Belgium

**Keywords:** *Botrytis cinerea*, *Malus* × *domestica (Borkh)*, mevalonate (MVA) pathway, ethylene, phytopathology, RNAseq, hot water dipping

## Abstract

Apple is typically stored under low temperature and controlled atmospheric conditions to ensure a year round supply of high quality fruit for the consumer. During storage, losses in quality and quantity occur due to spoilage by postharvest pathogens. One important postharvest pathogen of apple is *Botrytis cinerea*. The fungus is a broad host necrotroph with a large arsenal of infection strategies able to infect over 1,400 different plant species. We studied the apple-*B. cinerea* interaction to get a better understanding of the defense response in apple. We conducted an RNAseq experiment in which the transcriptome of inoculated and non-inoculated (control and mock) apples was analyzed at 0, 1, 12, and 28 h post inoculation. Our results show extensive reprogramming of the apple’s transcriptome with about 28.9% of expressed genes exhibiting significant differential regulation in the inoculated samples. We demonstrate the transcriptional activation of pathogen-triggered immunity and a reprogramming of the fruit’s metabolism. We demonstrate a clear transcriptional activation of secondary metabolism and a correlation between the early transcriptional activation of the mevalonate pathway and reduced susceptibility, expressed as a reduction in resulting lesion diameters. This pathway produces the building blocks for terpenoids, a large class of compounds with diverging functions including defense. 1-MCP and hot water dip treatment are used to further evidence the key role of terpenoids in the defense and demonstrate that ethylene modulates this response.

## Introduction

Apple (*Malus* × *domestica* Borkh.) is the third most produced fruit with a global total of about 83 million tons in 2017 (Food and Agriculture Organization [FAO], 2019). It is a seasonal fruit harvested in autumn and subsequently stored under low temperature and controlled atmospheric conditions to ensure year round supply of high quality fruit. During this storage, part of the fruit is lost due to spoilage by postharvest pathogens. An exploratory study we conducted for commercially grown and managed “Nicoter” apple found that the model necrotroph *Botrytis cinerea* had the third highest incidence (Naets et al., 2020).

*B. cinerea*, commonly known as gray mold, has a host range spanning over 1,400 species (Elad et al., 2016). A survey about fungal phytopathogens held by the journal *Molecular Plant Pathology* among researchers in the field considered *B. cinerea* the second most important fungal pathogen (Dean et al., 2012). Being a necrotroph, *B. cinerea* kills host cells for nutrition using a diverse arsenal of cell wall degrading enzymes (CWDEs), toxins and effectors. The fungus manipulates the plant’s defense response in his favor, inducing the hypersensitive response (HR) and programmed cell death (PCD), while suppressing other defense responses (Govrin and Levine, 2002; González et al., 2016).

Plants are under continuous threat of opportunistic and pathogenic microorganisms. During evolution, plants developed several mechanisms to recognize these attackers and mount a defense response. Recognition starts by surface-localized pattern recognition receptors (PRRs) or receptor-like proteins (RLPs) that bind well conserved microbe-associated molecular patterns (MAMPs) or damage-associated molecular patterns (DAMPs); the latter are formed from the plant itself by for instance breakdown of the cell wall. Recognition of MAMPs/DAMPs results in a signaling cascade that triggers innate immunity known as pathogen-triggered immunity (PTI) (Jones and Dangl, 2006; Boller and Felix, 2009). To circumvent PTI, pathogens can secrete effectors that prevent perception of MAMPs/DAMPs, e.g., ligands that bind chitin oligomers, or interfere with downstream signaling. This then results in effector-triggered susceptibility (ETS). As an answer to ETS, plants have evolved to recognize pathogen effectors through resistance (R) proteins, which are often nucleotide-binding leucine-rich repeat (NB-LRRs) proteins. Recognition of effectors then results in effector-triggered immunity (ETI), which often leads to cell death through the HR (Jones and Dangl, 2006; Boller and Felix, 2009). This classical zig-zag model does not hold for broad host necrotrophs (BHNs) such as *B. cinerea*, however. In this case, defense is quantitative (varying levels of susceptibility depending timing and intensity of an array of defense mechanisms) and variations in the level of susceptibility between species are thought to be linked to differences in innate immunity (Mengiste, 2012; Windram et al., 2012).

Most of this knowledge was established in model systems such as *Arabidopsis*, but a lot less research has been conducted on non-model systems. Transferring this knowledge becomes even trickier when studying fruit where for instance ethylene (ET) could have a dual role in defense and ripening. Still, understanding the defense response in fruit is fundamental, because losses due to pathogens can have an important economic impact.

Global transcriptome expression studies of domesticated apple have been carried out previously to investigate the interaction with the necrotrophic fungal pathogen *Pencillium expansum* (Vilanova et al., 2014; Ballester et al., 2017). Comparison with the incompatible *P. digitatum* showed that detoxification of reactive oxygen species (ROS) and upregulation of phenylpropanoid biosynthesis pathway play a crucial role (Vilanova et al., 2014). Comparing the response with that of the resistant *M. sieversii* demonstrated that a rapid activation of defenses could be a determinative factor in the outcome (Ballester et al., 2017).

In the current work, we wanted to deepen the knowledge about the defense response of apple. To this end, we present here the first global transcriptomic study of the *M.* × *domestica*-*B. cinerea* interaction in which we demonstrate the activation of PTI and secondary metabolism. We were able to correlate gene expression with infection success and thereby providing evidence for the importance of the early transcriptional activation of the mevalonate pathway that provides precursors for terpenoid biosynthesis. We further strengthen the case for the key role of terpenoids in defense through the use of 1-MCP and hot water dip (HWD) treatments, and demonstrate that ET plays a modulating role in this response.

## Materials and Methods

### Plant and Fungal Material

Apples of cultivar “Jonagored” (an economically important variety in Flanders) were harvested randomly at commercial maturity according to guidelines by the Flanders Centre of Postharvest Technology on 26 September 2016 from a field in Rillaar (Beglium, 50.963293°N, 4.879464°E). The trees were planted in the winter of 2014 and were grafted onto an M9 rootstock. The plant density was 1.5 m by 3.75 m. A standard integrated pest management schedule was followed, except that 1 month prior to harvest no fungicide treatments were carried out. Harvested fruit were stored at 1°C with regular atmosphere (RA) until 29 September 2016 when atmospheric conditions were changed to controlled atmosphere (CA; 1% O_2_ and 2.5–3.0% CO_2_). The experiment was started after a total of about 15 w of storage. Apples were transferred to 22°C with RA 1 day prior to the start of the experiment. The apples used in the experiment had an average mass of 266 ± 6.1 g (mean ± se, *n* = 108) and respiration rate of 159 ± 7.4 nmol kg^–1^ s^–1^ (mean ± se, *n* = 5). Other quality measures are given in Table 1 and indicate that apples had not ripened much since harvest where respiration rate was similar at levels of 165 ± 9.7 nmol kg^–1^ s^–1^ (mean ± se, *n* = 10). Fruit were not disinfected as our previous research indicated that this could result in a physiological response and damage to the fruit surface, both of them potentially able to bias the study results (Naets et al., 2018).

**TABLE 1:** Average firmness, total soluble solids (TSS) content and hue of the batch of “Jonagored” apples at harvest (26 September 2016) and the start of the experiment (12 January 2017).

Side	Firmness (N)	TSS (%)	Hue (°)
**Harvest**			
Shadow	84 ± 3.6	15.4 ± 0.38	79 ± 3.5
Sun	88 ± 1.8	16.6 ± 0.31	
**Start of experiment**			
Shadow	94 ± 2.7	16.5 ± 0.24	87 ± 2.6
Sun	98 ± 2.2	17.5 ± 0.23	

*Errors denote the standard error of the mean (Harvest: n = 20, Start of experiment: n = 15).*

A *B. cinerea* B05.10 isolate was kindly provided by Julia Schumacher (Institut für Biologie and Biotechnologie der Pflanzen). The fungus was cultured on potato dextrose agar in the dark. After 5–7 days when the colony reached the periphery of the petri dish the culture was exposed to near-UV light overnight to induce sporulation and subsequently put back in the dark for another 5–7 days. Spores were harvested by flooding the plates with 5 mL of sterile distilled water. The suspension was filtered through glass wool and washed twice with 12 g L^–1^ potato dextrose broth (PDB) in order to remove any remaining mycelia. Finally, the spore concentration was adjusted to 10^6^ conidia mL^–1^.

### RNAseq Experiment

Three wounds were made on the shadow side of the fruit at its equator with a depth of 10.5 mm and a diameter of 1.8 mm. This method had been shown to give reproducible infections. The side of the fruit was arbitrary, but important to fix, because previous research of us showed that the side of the fruit could have an effect on susceptibility to *B. cinerea* (Naets et al., 2019). Each wound was inoculated with either 2 μL of the spore suspension (which will be denoted as *B. cinerea* inoculated) or 12 g L^–1^ PDB (mock inoculated). The control consisted of samples from non-treated fruit (not wounded, not inoculated). The middle one of the three inoculated spots was sampled at specific time points post inoculation using a cork borer of size 8 (13.75 mm diameter). Samples were cut off at a depth of 15 mm and were immediately quenched in liquid nitrogen. The two outer spots were not sampled and, in the case of *B. cinerea* inoculated spots, were evaluated for lesion diameter 96 hpi (hours post inoculation) using a digital caliper. An example of a sampled apple at 96 hpi is given in Supplementary Figure 1.

Fruits were not treated (control), mock or *B. cinerea* inoculated. Immediately within the first 5 min after treatment (0), at 1, 12, and 28 hpi three biological replicates (apples) were sampled. This experiment was carried out in triplicate, thus giving 3 independent experiments with each 3 biological replicates per treatment per time point. The total number of samples available for sequencing was thus 36 (3 treatments ×x 3 replicates × 4 time points). Additionally, for the samples taken at 28 hpi, five biological replicates from each treatment were analyzed for metabolic changes by GC-MS and LC-MS to confirm transcriptomic results. The time point of 28 hpi was the earliest one where measurable differences in metabolite levels could reasonable be expected.

### RNA Extraction and Sequencing

Total RNA was extracted by adding 800 μL of CTAB extraction buffer (2% CTAB, 2% PVP40, 100 mM Tris-HCl (pH8), 25 mM EDTA, 2 M NaCl, 0.5 g L^–1^ spermidine, 1% β-mercaptoethanol) to 150–200 mg finely crushed tissue sample. Subsequently, 80 μL N-laurylsarcosine sodium salt (20%) and a glass bead were added, and the mixture was incubated for 10 min in a thermomixer set at 70°C and 1,400 rpm. Next, 900 μL chloroform:isoamylalcohol (24:1) was added and the samples were centrifuged at 21,500 G and 22°C for 10 min. The supernatant was transferred to the gDNA eliminator spin column of the RNeasy Plus Mini kit from Qiagen (Manchester, United Kingdom). From here on, the manufacturer’s guidelines for the kit were followed. Quality and concentration of the extracts were verified using a NanoDrop™ 2,000 (Leusden, Netherlands) and by gel electrophoresis. Equal amounts of RNA were pooled across experiments to obtain three unique replicates per treatment per time point. The pooled RNA was precipitated by adding 2 vol 100% ethanol and 0.1 vol 3 M sodium acetate (pH5.2–5.5), incubating at -80°C overnight, and centrifugating at 21,500 G. The precipitate was washed twice with 70% ethanol before being resuspended in nuclease free water. All A260/A280 and A260/A230 ratios were above 2. Pooled samples of with at least 4 μg RNA were sent to Polar Genomics (New York, United States) for sequencing. A stranded library was constructed for each sample with fragment lengths of 75 bp, as described by Zhong et al. (2011). Sequencing was performed on an Illumina Nextseq500 platform (San Diego, United States). Generated reads were cleaned up and subsequently aligned to the “Golden Delicious” (“Jonagold” is a cross of “Jonathan” and “Golden Delicious”, and “Jonagored” is a color mutant of “Jonagold”) apple genome (Daccord et al., 2017) and *B. cinerea* genome (Van Kan et al., 2017).

### RNAseq Analysis

Statistical analysis was carried out in R (R Development Core Team, 2011). Differential expression analysis was performed using the DESeq2 package (Love et al., 2014). For each time point, differentially expressed genes (DEGs) were identified for the comparison of *B. cinerea* inoculated with control, and *B. cinerea* inoculated with mock inoculated fruit. DEGs were filtered for those showing at least twofold up or downregulation and subsequently filtered for those that were differentially regulated in both comparisons. The resulting DEGs were considered significantly differentially expressed. Heat maps for a specific enzyme were created from all genes coding for isozymes exhibiting significant differential expression on at least one time point by taking a weighted mean of the log_2_ fold change (LFC) as described by the following formula.


$$L\,F\,C=\frac{\sum_{i}\left[\left|d_{i\,1}+d_{i\,2}\right|\cdot L\,F\,C_{i}\right]}{\sum_{i}\left|d_{i\,1}+d_{i\,2}\right|}$$

In this formula *d*_*i1*_ and *d*_*i2*_ are the difference in expression in transcripts per million (TPM) for the *i*-th isozyme between *B. cinerea* inoculated and control and *B. cinerea* inoculated and mock inoculated fruit, respectively.

### Enrichment Analysis

Enrichment of specific functions was analyzed using significant DEGs as test set, and all genes exhibiting at least 1 TPM on average in control and mock inoculated samples were used as reference. Gene ontology (GO) enrichment analysis was carried out in BLAST2GO (Götz et al., 2008). Additionally, enrichment of pathways listed on the Kyoto Encyclopedia of Genes and Genomes (KEGG) database (Kanehisa, 2000) was tested. Enrichment of KEGG database pathways was scripted in R by mapping genes to their pathways, performing a Fisher exact test for each pathway, and correcting the *p*-values for multiple hypothesis testing using the Benjamini-Hochberg procedure.

### Gas Chromatography Mass Spectrometry

Ground apple samples (15 appels, 28 hpi) were extracted with 1 mL of methanol at room temperature for 2.5 h, followed by centrifugation. Under vacuum, 450 μL of the supernatant was evaporated to dryness, and the obtained residue was derivatized for gas chromatography mass spectrometry (GC-MS) analysis by adding 10 μL of pyridine and 50 μL of N-Methyl-N-(trimethylsilyl) trifluoroacetamide (Sigma-Aldrich, Saint Louis, MO, United States). GC-MS analysis was carried out using a 7890B GC system equipped with a 7693A Automatic Liquid Sampler and a 7,250 Accurate-Mass Quadrupole Time-of-Flight MS system (Agilent Technologies, Santa Clara, CA, United States). 1 μL of the sample was injected in splitless mode with the injector port set to 280°C. Separation was achieved with a VF-5 ms column (30 m × 0.25 mm, 0.25 μm; Varian CP9013; Agilent Technologies) with helium carrier gas at a constant flow of 1.2 mL min^–1^. The oven was held at 80°C for 1 min post-injection, ramped to 280°C at 5°C min^–1^, held at 280°C for 5 min, ramped to 320°C at 20°C min^–1^, held at 320°C for 5 min, and finally cooled to 80°C at 50°C min^–1^ at the end of the run. The MSD transfer line was set to 280°C and the electron ionization energy was 70 eV. Full EI-MS spectra were recorded between m/z 50–800 at a resolution of > 25,000 and with a solvent delay of 7.8 min. The resulting GC-MS chromatograms were converted to SureMass format, deconvoluted, and metabolites corresponding to the deconvoluted spectra were identified using the MassHunter Unknowns Analysis software package (Agilent Technologies) and the NIST 17 mass spectral library. A match factor of 75.0 was used as cut-off for compound identification. For each metabolite, a unique quantifier ion was chosen and used for quantification using the MassHunter Quantitative Analysis (for Q-TOF) software package (Agilent Technologies). In total, 285 metabolites were detected and quantified, of which 117 (41%) were identified based on their EI-MS spectra. Data post-processing was done with MetaboAnalyst 3.0.

### Liquid Chromatography Mass Spectrometry

The ground apple samples were extracted with 1 mL of methanol at room temperature for 2.5 h, followed by centrifugation. Under vacuum, 450 μL of the supernatant was evaporated to dryness, and the obtained residue was re-suspended in 100 μL cyclohexane/100 μL MilliQ water. The samples were centrifuged, and 90 μL of the lower water phase was transferred to a 96-well plate for LC-MS analysis. LC-MS analysis was performed on an ACQUITY UPLC I-Class system (Waters) consisting of a binary pump, a vacuum degasser, an autosampler, and a column oven. Chromatographic separation was carried out on an ACQUITY UPLC BEH C18 (150 × 2.1 mm, 1.7 μm) column from Waters, and temperature was maintained at 40°C. A gradient of two buffers was used: buffer A (99:1:0.1 water:acetonitrile:formic acid, pH 3) and buffer B (99:1:0.1 acetonitrile:water:formic acid, pH 3), as follows: 95% A for 0.1 min decreased to 50% A in 30 min, decreased to 0% from 30 to 41 min. The flow rate was set to 0.35 mL min^–1^, and the injection volume was 15 μL. The UPLC system was coupled to a Synapt G1 Q-TOF hybrid mass spectrometer (Waters). The LockSpray ion source was operated in negative electrospray ionization mode under the following specific conditions: capillary voltage, 3 kV; reference capillary voltage, 2.5 kV; cone voltage, 37 V; extraction cone, 3.5; source temperature, 120 °C; desolvation gas temperature, 400 °C; desolvation gas flow, 550 L h^–1^; and cone gas flow, 50 L h^–1^. The collision energy for full MS scan was set at 6 eV, for DDA MSMS ramped from 10 to 20 eV for low mass and from 20 to 45 eV for high mass. Mass range was set from 100 to 1,200 Da. Nitrogen (greater than 99.5%) was employed as desolvation and cone gas. Leucin-enkephalin (250 pg μL^–1^ solubilized in water:acetonitrile 1:1 [v/v], with 0.1% formic acid) was used for the lock mass calibration, with scanning every 1 min at a scan time of 0.1 s. Centroid data were recorded through Masslynx Software v4.1 (Waters). Data processing was performed with Progenesis QI software version 2.4 (Waters), data post-processing was done with MetaboAnalyst 3.0.

### 1-Methylcyclopropene and Hot Water Dipping Experiments

Apples from the same trees as the RNAseq experiment were harvested on 18 September 2018 and were either dipped the same day in water of 50°C for a duration of 5 min (HWD5) or exposed the next day to 625 nL L^–1^ 1-methylcyclopropene (1-MCP) at 1°C for 24 h. Fruit were subsequently transferred to the same CA conditions as described above. At regular time points, a sample of fruit was taken out of CA storage to carry out an inoculation experiment as described above. Samples for gene expression analysis were taken from both the sun and shadow side of non-inoculated fruit at harvest and after 29 w of CA storage for HWD5 treatment, and after 12 and 29 w of CA storage for 1-MCP treatment. It was decided to take two time points to evaluate if the fruit response diminished over time. Samples were taken with a cork borer of size 8 (13.75 mm diameter) and were divided into peel (0–3 mm) and flesh (4–15 mm) tissue.

### Quantitative Reverse Transcription Polymerase Chain Reaction

cDNA was synthesized using the Quantitect Reverse Transcription kit (Qiagen, United Kingdom) following the manufacturer’s specifications. RT-qPCR reactions were carried out with SsoAdvanced™ Universal SYBR Green Supermix (Bio-rad, Belgium) and cycling settings: 95°C for 10 min followed by 40 cycles of 95°C for 10 s and 63°C for 30 s. As a reference we used ubiquitin-conjugating enzyme E2 (*UBC*) which was shown to have a stable expression in apple under a plethora of postharvest conditions (Storch et al., 2015). Primer sequences used are listed in Supplementary Table 1.

### Temperature Simulations

Thermal simulations of heating and cooling of a single apple were performed in ANSYS 19.0 CFX (ANSYS, Inc., Canonsburg, Pennsylvania, United States). Fourier’s second law of transient conduction was solved with constant uniform thermal properties. The thermal conductivity, specific heat and density of apple were 0.3972 W m^–1^ K^–1^, 3,455 J kg^–1^ K^–1^ and 800 kg m^–3^, respectively (Gruyters et al., 2018). For heating in water, it was assumed that the surface heat transfer coefficient was infinitely large, such that a Dirichlet boundary condition with a constant surface temperature of 50°C was implemented. During the cooling in air at 20°C, it was assumed that the surface heat transfer coefficient was 20 W m^–2^°C^–1^. The model equations were solved on the geometry of a “Jonagold” apple (a mutant of “Jonagored” for which a geometry was available) with an approximate diameter of 7 cm, of which a CAD surface was generated based on an X-ray computed tomography scan (Gruyters et al., 2018). A tetrahedral finite volumes mesh with 118,221 elements and 22,505 nodes was used for the numerical solution of the model in ANSYS. Transient simulations were performed with a second order backward Euler scheme with a time step of 10 s during heating and 30 s during cooling. The iterative convergence criterion was 10^–7^ RMS of the residual of the energy equation with a maximum of 10 iterations per time step. CPU time was 3 min 30 s for 5 min of heating on an Intel Xeon CPU E5-2630 0 @ 2.30 GHz. The following conditions were simulated: heating in water for 3, 4 and 5 min at 50°C, each time followed by air cooling at 20°C for 1 h.

## Results

### RNA Sequencing

A total of 506,119,623 reads were generated using an Illumina Nextseq500 platform. After cleanup of the reads, a total of 470,274,412 high quality reads remained, averaging about 13 million reads per sample. An overview of the number of reads per sample and the amounts removed at each cleanup step is given in Supplementary Table 2. On average about 93 and 0.42% of the genes could be mapped to the *M.* × *domestica* and *B. cinerea* genome, respectively. The fraction of reads assigned to *B. cinerea* increased with time after inoculation and reached an average of 4.37% at 28 hpi.

Differential regulation of gene expression was tested using DESeq2 in R (Love et al., 2014). A total of 6,588 genes were identified as significantly upregulated of which 3,876 exhibited a fold change of more than 2 on at least one time point, and a total of 3,616 genes were identified as significantly downregulated of which 899 had a fold change of at least 2 on at least one time point (Table 2).

**TABLE 2:** Differential expression analysis using DESeq2 in R.

Time point (hpi)	*Botrytis* inoculated vs. control	*Botrytis* inoculated vs. mock inoculated	Overlap	Abs (LFC) > 1
0	167 + 60	71 + 5	67 + 0	65 + 0
1	539 + 13	1 + 0	0 + 0	0 + 0
12	2,636 + 316	1,561 + 32	1,501 + 8	1,100 + 0
28	8,016 + 5,830	7,112 + 4,364	6,573 + 3,612	3,863 + 899
Overall (0, 1, 12, 28)	8,199 + 5,981	7,136 + 4,389	6,588 + 3,616	3,876 + 899

*For each time point the number of genes up + downregulated are displayed for the comparison of Botrytis inoculated samples with the control, and Botrytis inoculated samples with mock inoculated samples. Additionally, the number of up + downregulated genes that overlap between those two comparisons is given, and the number of these genes that exhibited an absolute log_2_ fold change of at least 1 compared to mock inoculation. Up + downregulated.*

**FIGURE 1:**
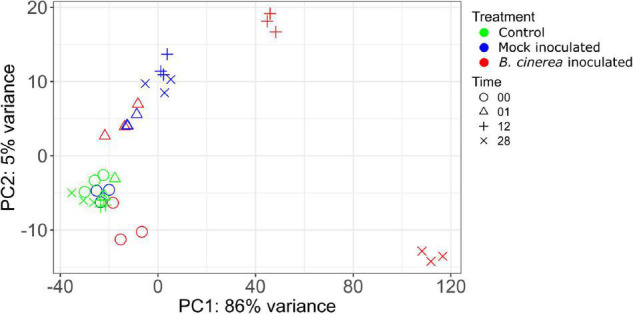
Biplot of a PCA performed on the regularized log transformed counts of the top 500 most variable genes showing the position of each sample on PC1 and PC2.

A principal component analysis (PCA) on the 500 most variable (standard setting of plotPCA function from DESeq2) apple genes after regularized log transformation demonstrates that the replicates had comparable results (Figure 1). Furthermore, plotting of replicate samples against one another (Supplementary Figures 2–5) showed high correlations averaging 98.8 ± 0.001% (se, *n* = 36). This proves the reproducibility of the assay and sequencing technique used.

The PCA biplot (Figure 1) shows that control samples of all time points cluster closely together, signifying that control samples did not display transcriptional changes on the same scale as the mock or *Botrytis* inoculated samples for the duration of the experiment. Furthermore, changes due to mock treatment were also much smaller than due to *Botrytis* inoculation. Samples taken immediately after administering the treatment were designated as 0 hpi, in practice, they were taken about 1–5 min after inoculation. The mock inoculated samples of 0 hpi were very similar to the control samples, while the *Botrytis* inoculated samples of 0 hpi were already clearly distinct from the control (Figure 1). This demonstrates that some responses upon pathogen recognition were very rapid. Mock and *Botrytis* inoculated samples of 1 hpi lie closely together in the plot, which indicates that the early wound and defense response strongly overlapped. This is reflected in the fact there were no DEGs at 1 hpi.

For a more in depth analysis, annotation data was obtained from the Genome Database for Rosaceae (GDR; Jung et al., 2004).^1^ This database contains a list of KEGG ortholog annotations for about 24.6% of the apple genome. This translated to about 19.5% annotation of the expressed genes in our data (mean tpm > 1) and 26.3% of the DEGs. Also a list with GO annotation for 59.2% of the apple genome was obtained. This amounted to 63.5% of expressed genes and 69.2% of the DEGs. In the following when we talk about pathways, we mean the transcriptional regulation of these pathways.

**FIGURE 2:**
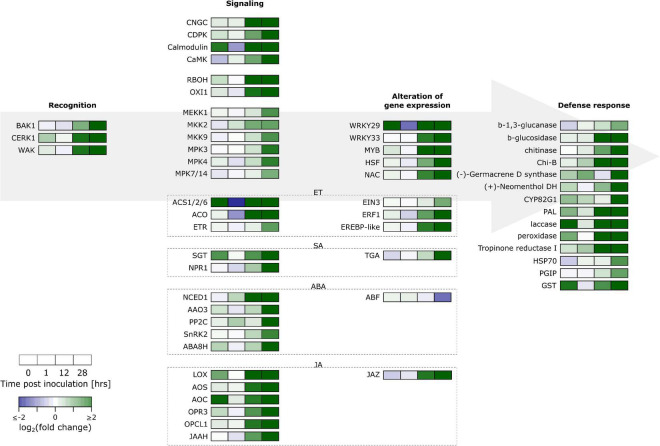
Overview of gene expression responses of “Jonagored” apple to *B. cinerea* inoculation. BAK1, Brassinosteroid insensitive-associated receptor kinase 1; CERK1, Chitin elicitor receptor kinase 1; WAK, Wall-associated kinase-like; CNGC, Cyclic nucleotide gated channel; CDPK, Calcium-dependent protein kinase; CAMK, Ca^2+^/calmodulin-dependent protein kinase; RBOH, Respiratory burst oxidase homolog; OXI1, Oxidative signal-inducible1 serine/threonine-protein kinase; MEKK, Mitogen-activated protein kinase kinase kinase; MKK, Mitogen-activated protein kinase kinase; MPK, Mitogen-activated protein kinase; ACS1/2/6, 1-Aminocyclopropane-1-carboxylate synthase 1/2/6; ACO, 1-Aminocyclopropane-1-carboxylate oxidase; ET, Ethylene; ETR, ET receptor; EIN3, ET-insensitive protein 3; ERF1, ET-responsive transcription factor 1; EREBP, ET-responsive element binding protein; SA, Salicylic acid; SGT SA glucosyltransferase; NPR1, Non-expresser of PR genes 1; TGA, TGACG binding factor 1; ABA, Abscisic acid; NCED1, 9-Cis-epoxycarotenoid dioxygenase 1; AAO3, Abscisic aldehyde oxidase; PP2C, Protein phosphatase 2C; SnRK2, Serine/threonine-protein kinase SRK2; ABA8H, ABA 8**’**-hydroxylase; ABF, ABA responsive element binding factor; JA, Jasmonic acid; LOX, Lipoxygenase; AOS, Allene oxide synthase; AOC, Allene oxide cyclase; OPR3, 12-Oxo-cis-10,15-phytodienoic acid reductase; OPCL1, 3-Oxo-2-(cis-2**’**-pentenyl)-cyclopentane-1-octanoic acid-8:0 CoA ligase; JAAH, Jasmonoyl-L-amino acid hydrolase; JAZ, JA ZIM domain protein; WRKY, Transcription factor WRKY; MYB, Myeloblastosis oncogene transcription factor; HSF, Heat shock transcription factor; NAC, NAM, ATAF1/2, and CUC transcription factor; Chi-B, basic endochitinase B; (+)-Neomenthol DH: (+)-Neomenthol dehydrogenase; CYP82G1, Cytochrome P450 family 82 subfamily G polypeptide 1; PAL, Phenylalanine ammonia-lyase; HSP70, Heat shock protein 70; PGIP, Polygalacturonase inhibiting protein; GST, Glutathione S-transferase.

For our analysis, we were guided by the results from an enrichment analysis of GO terms and KEGG pathways (Supplementary Figure 6 and Supplementary Tables 3, 4). This revealed upregulation of secondary metabolism, especially for phenylpropanoids and terpenoids, and a role for hormone regulation. A summary of key responses is given in Figure 2 and Supplementary Table 5 where a distinction is made between recognition, signaling, alteration of gene expression and defense response. Some key aspects will be discussed in brief below.

### Pathogen Recognition

Induction of PTI through recognition of M/DAMPs is mediated by PRRs and RLPs. The largest family of plant receptors are receptor-like kinases (RLKs), which are PRRs. One such RLK is the WALL-ASSOCIATED KINASE (WAK) that detects oligogalacturonides (OGs) (Decreux and Messiaen, 2005), DAMPs formed during breakdown of the plant cell wall by the fungus. Because no WAKs were annotated in the KEGG ortholog list obtained for apple from the GDR (Jung et al., 2004), we extracted WAK protein sequences from the Apple Gene Function and Gene Family Database (AppleGFDB, Zhang et al., 2013). We BLASTp searched these sequences in the new apple genome and used an *E*-value cutoff of 10^–100^. This resulted in 47 WAK genes. Of these 47, we retrieved 17 in our DEG set. Chitin is the main component of the fungal cell wall. Chitin oligosaccharides can be detected by the plant’s CHITIN ELICITOR RECEPTOR KINASE 1 (CERK1) (Miya et al., 2007). We retrieved four genes in the apple genome that were annotated with CERK1 and three of these were significantly upregulated in *B. cinerea* inoculated samples. In apple there are five genes annotated as the LEUCINE RICH REPEAT RECEPTOR-LIKE KINASE (LRR-RLK) BAK1. One of these, *MD08G1221700*, was significantly upregulated in our data (Supplementary Table 5).

### Defense Signaling and Transcriptional Reprogramming

One of the earliest responses to pathogen recognition that occurs within the first minutes is the establishment of Ca^2+^ influx into the cell (Reddy et al., 2011), established by CYCLIC NUCLEOTIDE-GATED CHANNELS (CNGCs). Our data contains 11 out of 40 differentially regulated CNGCs. The Ca^2+^ signal is transduced by sensor relays, e.g., CALMODULIN (CaM; 5/16 significantly upregulated), and sensor responders, e.g., CALCIUM-DEPENDENT PROTEIN KINASE (CDPK; 6/32 differentially regulated) and Ca^2+^/CaM-DEPENDENT PROTEIN KINASEs (CaMKs; 1/2 significantly upregulated). CDPKs can phosphorylate RESPIRATORY BURST OXIDASE HOMOLOGs (RBOHs; 1/8 significantly upregulated) and TFs (Reddy et al., 2011).

ROS are formed quickly upon wounding or pathogen challenge. They can have a direct role in stopping the pathogen by being directly toxic or by creating barriers through oxidative cross-linking of lignin precursors and cell wall glycoproteins (Torres, 2010). ROS also play an indirect role activating defense responses as second messengers. This is achieved by activating MITOGEN-ACTIVATED PROTEIN KINASE (MAPK) cascades. To achieve full activation of the MAPKs MPK3 and MPK6, OXI1 is required (Rentel et al., 2004). The apple genome has two genes that are annotated with OXI1 of which 2 were significantly upregulated. Two MPK3 and two MPK6 genes are annotated in the apple genome, and of these, respectively, one and zero genes were upregulated. The BAK1, CERK1 and WAK receptors also make use of MAPK signaling cascades that go through MAPK KINASE KINASE (MAPKKK), MAPK KINASE (MAPKK) and finally MAPK. Our expression set has two MAPKKK DEGs annotated with MEKK1, two MAPKK DEGs annotated with MKK2 and MKK9, and five MAPK DEGs annotated with MPK3, MPK4 (3 genes) and MPK7/14 (Supplementary Table 5).

Plant hormones play an important role in modulating plant responses to pathogens. Defense responses are regulated by the two major players salicylic acid (SA) and jasmonic acid (JA), while other hormones such as ET and abscisic acid (ABA) play a role in fine tuning the response (Pieterse et al., 2012; Bürger and Chory, 2019). In general, SA signaling confers resistance to (hemi-) biotrophic pathogens, and JA and ET signaling leads to resistance against necrotrophic pathogens. We found significant upregulation of JA biosynthetic genes coding for LIPOXYGENASE (LOX), ALLENE OXIDE SYNTHASE (AOS), ALLENE OXIDE CYCLASE (AOC), OXO-CIS-10,15-PHYTODIENOIC ACID REDUCTASE (OPR3) and 3-OXO-2-(CIS-2′-PENTENYL)-CYCLOPENTANE-1-OC TANOIC ACID-8:0 CoA LIGASE (OPCL1). SA biosynthesis can be achieved through the ISOCHORISMATE SYNTHASE (ICS) pathway or the PHENYLALANINE AMMONIA-LYASE (PAL) pathway. We found one gene annotated with ICS that was not differentially regulated. All four genes annotated as PAL were significantly upregulated. SA causes a redox change through activation of thioredoxins which leads to monomerization of NON-EXPRESSOR OF PR1 (NPR1), thereby facilitating its translocation to the nucleus. NPR1 then interacts with TGA transcription factors to effectuate changes in gene expression. DESeq2 analysis showed upregulated DEGs for NPR1 and TGA. Two of the four differentially expressed TGA genes identified by DESeq2 were upregulated and two were downregulated. Also biosynthesis of ET was upregulated with genes coding for 1-AMINOCYCLOPROPANE-1-CARBOXYLATE (ACC) SYNTHASE 1/2/6 (ACS1/2/6), ACC OXIDASE (ACO). ET signaling occurs by perception of ET by the ET RECEPTOR (ETR) localized in the endoplasmic reticulum. Binding of ET to the receptor causes inactivation of the ETR-CTR1 (CONSTITUTIVE TRIPLE RESPONSE 1) complex which leads to derepression of ET INSENSITIVE PROTEIN 2 (EIN2). EIN2 activates the transcription factor EIN3, which in turn causes transcription of other transcription factors such as ERF1. Our data shows upregulation of genes coding for ETR, EIN3, and ERF1 (Supplementary Table 5). An overview is provided in Supplementary Figure 7.

Signaling upon pathogen recognition should eventually lead to transcriptional reprogramming by activating or repressing transcription factors. Our data contained DEGs encoding transcription factors of several different families such as 5 WRKYs, 21 MYBs (myeloblastosis oncogene), 12 HSFs (heat shock factors) and 40 NACs (NAM, ATAF1/2, and CUC) (Supplementary Table 5).

### Transcriptional Reprogramming of the Metabolism for Defense

Our data provides evidence (Supplementary Table 6) in support that primary metabolism is reprogrammed to provide energy for the defense response (Bolton, 2009). Photosynthesis was significantly downregulated with 6/17 genes coding for components of photosystem I, 4/32 genes coding for components of photosystem II, 4/10 genes involved in light-harvesting complex I, and 6/20 genes involved in light-harvesting complex II significantly downregulated. Oxidation of fatty acids was upregulated (Supplementary Figure 8) with genes coding for LONG-CHAIN ACYL-CoA SYNTHETHASE (2/12), ACYL-CoA OXIDASE (2/6), ENOYL-CoA HYDRATASE/3-HYDROXYACYL- CoA DEHYDROGENASE (2/5), ACETYL-CoA ACYLTRANSFERASE 1 (2/3), and ACETYL- CoA C-ACETYLTRANSFERASE (2/3). Glycolytic activity was upregulated (Supplementary Figure 9) with DEGs for at 6-PHOSPHOFRUCTOKINASE 1 (PFK-1; 3/11), HEXOKINASE (1/9) and PYRUVATE KINASE (2/17). The final product of the glycolysis, pyruvate, is converted into acetyl-CoA by PYRUVATE DEHYDROGENASE (PDH). PDH is a complex of three enzymes (E1, E2, and E3) and E1 consists of two subunits (E1a and E1b). DEGs were identified for all of these components with 1 out of 3, 2 out of 3, 2 out of 9 and 1 out of 3 for E1a, E1b, E2, and E3, respectively. PDH is regulated by phosphorylation and dephosphorylation of E1a, respectively, inactivating and activating the complex. No DEGs were identified for PDH KINASE, while 8 out of 13 PDH PHOSPHATASE genes were significantly upregulated. Acetyl-CoA forms a crossroad in plant metabolism. This molecule is formed from oxidation of glucose, fatty acids and amino acids, and can be used as building block for biosynthesis of fatty acids, triglycerides, phospholipids, steroids and terpenoids. Acetyl-CoA can also be further oxidized in the tricarboxylic acid (TCA) cycle to yield precursors for the oxidative phosphorylation, thereby contributing to energy production. Our data shows significant upregulation of *citrate synthase, aconitate hydratase*, and *isocitrate dehydrogenase* indicating that the TCA cycle is upregulated.

**FIGURE 3:**
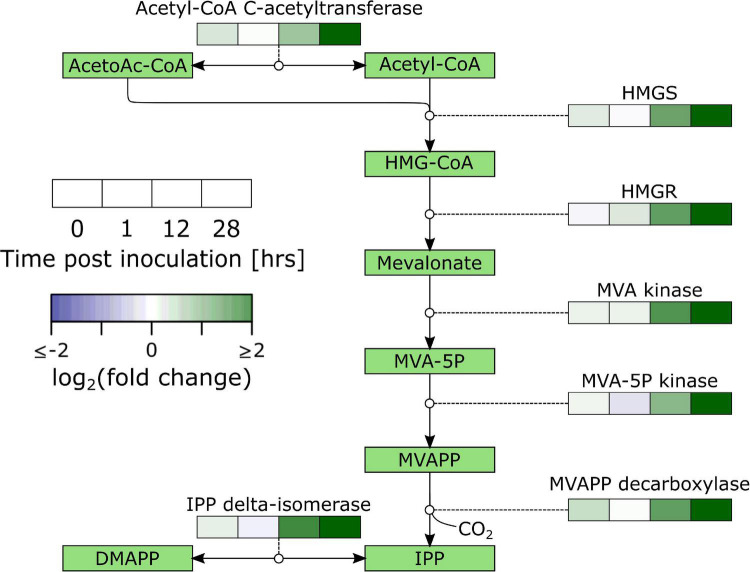
Overview of the regulation of the mevalonate pathway in *B. cinerea* inoculated apples. Heat maps were constructed from genes with significant differential expression. AcetoAc-CoA, Acetoacetyl-CoA; HMG-CoA, Hydroxymethylglutaryl-CoA; HMGS, HMG-CoA synthase; MVA, Mevalonate; MVAPP, Mevalonatepyrophosphate; IPP, Isopentylpyrophosphate; DMAPP, Dimethylallylpyrophosphate.

Secondary metabolism denotes pathways that are not directly involved in vital life functions and entails a wide variety of metabolic pathways with diverging functions. One of them is the production of plant defense compounds including terpenoids and phenylpropanoids. Biosynthesis of terpenoids from acetyl-CoA starts by biosynthesis of the isoprenoid precursor isopentenyl pyrophosphate (IPP) through the mevalonate (MVA) pathway (cytosol; biosynthesis of sesquiterpenoids and triterpenoids; Figure 3 and Supplementary Table 6) or the 2-C-Methyl-D-erythritol 4-phosphateZ (MEP) pathway (chloroplasts; biosynthesis of monoterpenoids, and diterpenoids). Our data shows a very distinct upregulation of the MVA pathway with significant upregulation of 2/2 HYDROXYMETHYLGLUTARYL-CoA (HMG-CoA) SYNTHASE (HMGS), 1/4 HMG-CoA REDUCTASE (HMGR), 2/2 MVA KINASE, 2/2 PHOSPHOMEVALONATE (MVA-5P) KINASE, and 2/2 MEVALONATEPYROPHOSPHATE (MVAPP) DECARBOXYLASE. The data suggests that IPP is further converted into sesquiterpenoids and triterpenoids with significant differential regulation of *farnesyl disphosphate* (FPP) *synthase* (*FPPS*; 4/4), FPP *farnesyltransferase* (*FPPFT*; 2/2), *squalene monooxygenase* (*SMO*; 10/15), *(-)-germacrene D synthase* (1/6), *β-amyrin synthase* (6/7) and *β-amyrin 28-monooxygenase* (8/14).

**FIGURE 4:**
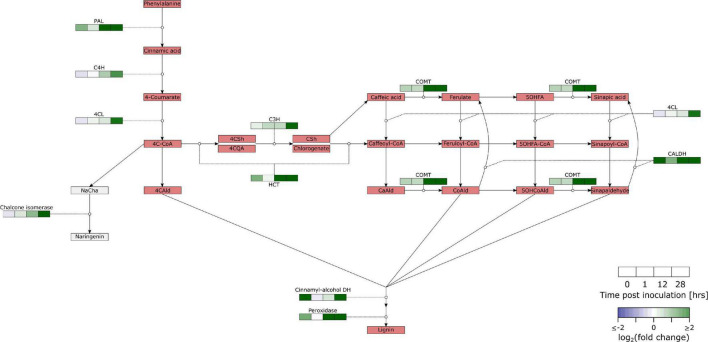
Overview of the regulation of the phenylpropanoid pathway in *B. cinerea* inoculated apples. Heat maps were constructed from genes with significant differential expression. PAL, Phenylalanine ammonia-lyase; C4H, Trans-cinnamate 4-monooxygenase; 4CL, 4-Coumarate:CoA ligase; 4C-CoA, 4-Coumaroyl-CoA; C3H, 4-Coumarate 3-hydroxylase; HCT, Hydroxycinnamoyl-CoA shikimate:quinate hydroxycinnamoyl-transferase; COMT, Caffeic acid/5-hydroxyferulic acid O-methyltransferase; CALDH, Coniferyl-aldehyde dehydrogenase; 4CAld, 4-Coumaraldehyde; NaCha, Naringenin chalcone; 4CSh, 4-Coumaroyl shikimic acid; 4CQA, 4-Coumaroyl quinic acid; CSh, Caffeoyl shikimate; CaAld, Caffeylaldehyde; CoAld: Coniferaldehyde; 5OHFA, 5-Hydroxyferulic acid; 5OHCoAld, 5-Hydroxyconiferylaldehyde. Another class of secondary metabolic compounds are alkaloids. This is a diverse group of nitrogen containing molecules, often with a function in plant defense. Our expression set suggests that biosynthesis of tropane alkaloids is upregulated in *B. cinerea* infected tissue. ARGININE DECARBOXYLASE (AGD) was significantly upregulated (2/2). AGD converts Arginine into agmatine, which can be further converted into putrescine and finally tropinone. Our data contained 9 DEGs for TROPINONE REDUCTASE I (TR-I; 16 genes) which reduces tropinone to tropine.

Phenylpropanoids are biosynthesized from phenylalanine (Figure 4 and Supplementary Table 6). The key step in this pathway is the first conversion by PAL **(Dixon et al., 2002)**. All of the four genes annotated with PAL were significantly upregulated in our data. Our data also shows differential regulation of C4H (TRANS-CINNAMATE 4-MONOOXYGENASE; 2/3), 4CL (4-COUMARATE:COA LIGASE; 2/7), C3H (4-COUMARATE 3-HYDROX YLASE; 2/3), HCT (4/17), COMT (CAFFEIC ACID/5-HYDROXYFERULIC ACID O-METHYLTRANSFERASE; 8/18), CALDH (CONIFERYL-ALDEHYDE DEHYDROGENASE; 1/5), CINNAMYL-ALCOHOL DEHYDROGENASE (10/20), PEROXIDASE (19/102), LACCASE (11/47), and CHALCONE ISOMERASE (CHI; 3/7).

Other metabolic responses detected in our data were differential regulation of cell wall metabolism with DEGs coding for 1,3-β-GLUCANASE (1/8), β-GLUCOSIDASE (8/46), CHITINASE (7/19), BASIC ENDOCHITINASE B (1/3) and POLYGALACTURONASE (PG) INHIBITING PROTEINs (PGIPs; 2/4) (Supplementary Table 5).

We attempted to verify some secondary metabolites by untargeted metabolomics. To this end, 5 of the sequenced samples of each treatment from 28 hpi were analyzed with GC-MS and LC-MS (Supplementary Tables 8, 9). GC-MS analysis found significant accumulation of D-galacturonic acid, galactaric acid and glycerol, probably linked to pectin breakdown by *B. cinerea* polygalacturonases. Otherwise, almost no significant differences were found, likely due to this time point being too early for detecting significant metabolic changes.

### Effectiveness of the Defense Response

In our experiment we sampled the middle of three inoculated spots. The outer spots were not sampled and the lesion diameter of these spots was recorded at 96 hpi. From other experiments we know that the correlation of the lesion diameter of the middle spot with the average of the outer two spots is about 88% (*p* < 0.001; Supplementary Figure 10). This allows us to correlate gene expression at a specific time point of sampling with later infection success, measured as lesion diameter at 96 hpi.

**FIGURE 5:**
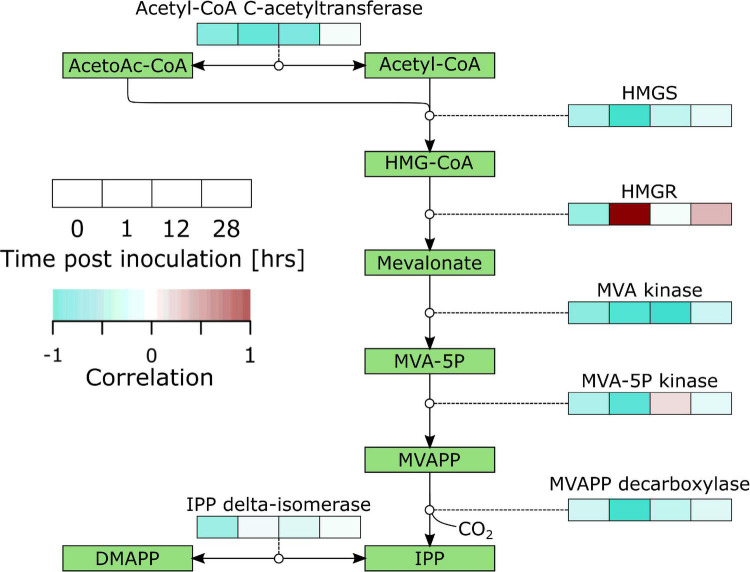
Overview of correlations of the mevalonate pathway gene expression with lesion diameter at 96 hpi in *B. cinerea* inoculated apples. Heat maps were constructed from genes with significant differential expression. AcetoAc-CoA, Acetoacetyl-CoA; HMG-CoA, Hydroxymethylglutaryl-CoA; HMGS, HMG-CoA synthase; MVA, Mevalonate; MVAPP, Mevalonatepyrophosphate; IPP, Isopentylpyrophosphate; DMAPP, Dimethylallylpyrophosphate.

We analyzed the correlations between gene expression and infection severity at 96 hpi for the discussed pathways and found a striking image for the MVA pathway. This pathway showed a mostly negative correlation with infection success (i.e., higher expression led to lower susceptibility) and strong negative correlations occurred mostly at the early time points (Figure 5).

### Effect of 1-Methylcyclopropene and HWD5 Treatment on Fruit Defense

**FIGURE 6:**
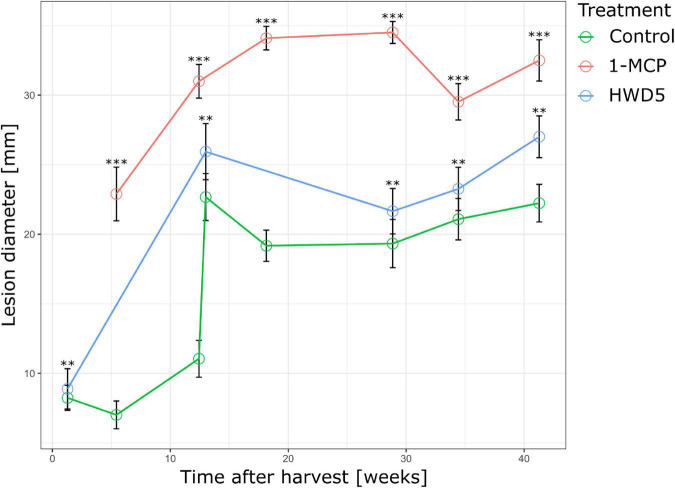
Lesion diameter 96 hpi of pathogenicity assay performed at various time points throughout CA storage for control, HWD5 and 1-MCP treated apples inoculated with *B. cinerea*. Error bars denote the standard error of the mean (*n* ≥ 10). Significant differences are indicated by an asterisk. ^**^p < 0.01 ^***^p < 0.001.

In a next series of experiments we wanted to investigate the effect of a HWD of 5 min (HWD5), or 1-MCP treatment on the defense of apple against *B. cinerea*, and this as a function of time in CA storage. We carried out our pathogenicity assay on treated fruit after different durations of CA storage and found that both 1-MCP and HWD5 treatment lead to a significant increase in lesion diameter after 96 hpi (Figure 6).

**FIGURE 7:**
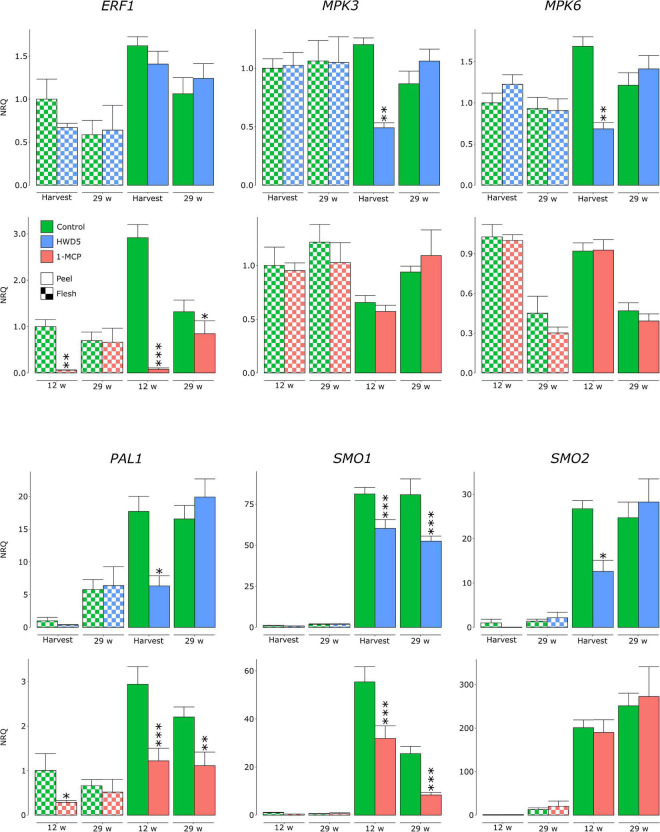
Normalized relative quantities of genes analyzed in flesh and peel samples of “Jonagored” apples belonging to control, HWD5 or 1-MCP treatment. Samples were analyzed at harvest and after 29 w of CA storage (control, HWD5) or after 12 and 29 w of CA storage (control, 1-MCP). Error bars denote the standard errors of at least 6 biological replicates. Statistically significant differences with the control are denoted with asterisks: *p < 0.05 ^**^p < 0.01 ^***^p < 0.001.

**FIGURE 8:**
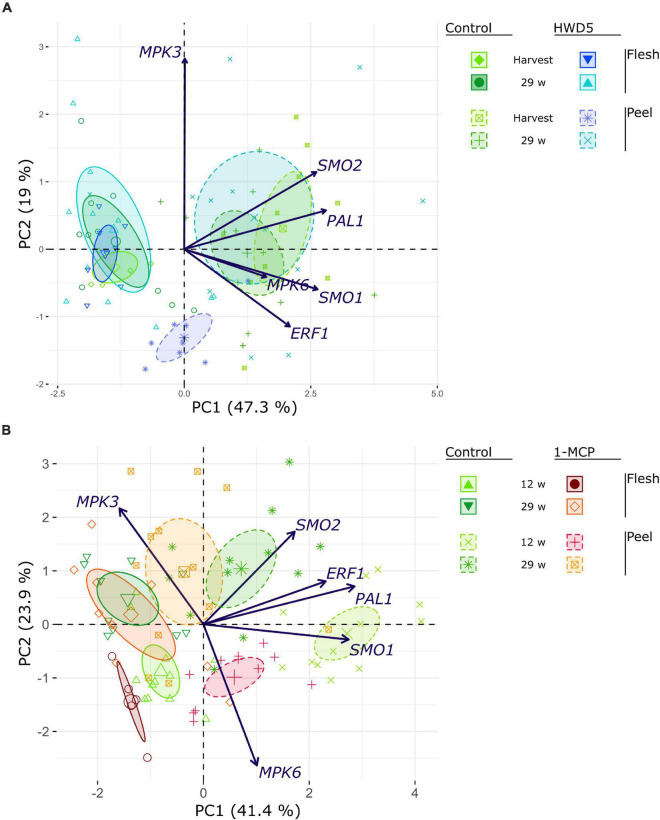
PCA biplot of gene expression analyses performed on **(A)** control and HWD5 at harvest or after 29 w of CA storage, or **(B)** control and 1-MCP treated apples that had been stored with CA 13 or 29 w. Ellipses denote 95% confidence intervals.

We analyzed expression of *MPK3*, *MPK6*, *ERF1*, *PAL1*, *SMO1*, and two *SMO* homologs (denoted *SMO1* and *SMO2*) in peel and flesh tissue of control, HWD5 and 1-MCP treated fruit (Figure 7 and Supplementary Table 10). HWD5 treatment caused only significant reductions in the peel. Expression of *MPK3*, *MPK6*, *PAL1*, *SMO1* and *SMO2* was affected at harvest. After 29 w of CA storage, only SMO1 still had a significantly lower expression compared to the control. 1-MCP treatment was able to also affect gene expression in flesh tissue, significantly reducing *ERF1* and *PAL1*, but only at 12 w in CA storage. In the peel, expression of *ERF1*, *PAL1* and *SMO1* was significantly reduced compared to the control at both 12 and 29 w. A PCA was performed on this expression data. The result demonstrates that peel has a clearly distinct expression pattern from flesh tissue and also reacts differently to the treatments (Figure 8).

**FIGURE 9:**
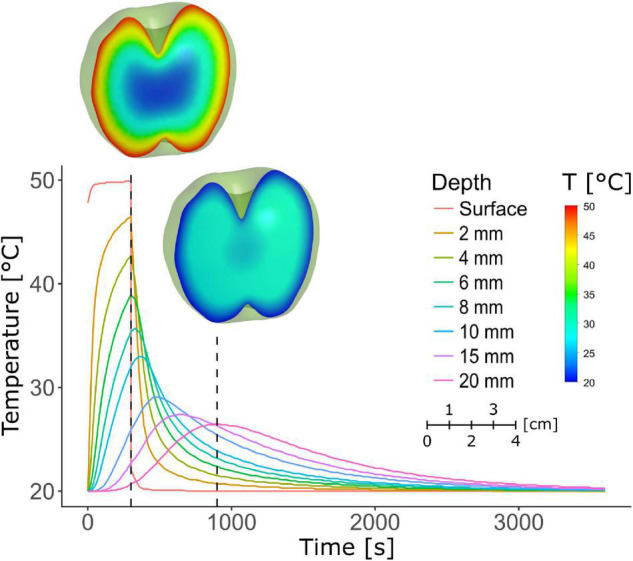
Simulation of the temperature profile in an apple as a function of time during and after a 50°C HWD of 5 min. The first dashed line marks the end of the 5 min treatment, the second marks 15 or 10 min of air cooling after the treatment. Fruit heatmaps corresponding to the dashed lines show the temperature profile in the fruit at those time points.

The temperature profiles of the fruit during and after the HWD5 treatment were simulated (Figure 9). The fruit surface rapidly reaches 50°C, but the temperature shows a strong downward gradient along the radius toward the core. Diffusion of heat causes the inside of the fruit to reach its maximum temperature only after 5 min of dipping. For example, at a radial depth of 15 mm the temperature reaches 25.9°C at the end of the 5 min HWD, but still increases to a maximum of 29.1°C after the treatment.

## Discussion

### Inoculation With *Botrytis cinerea* Caused Extensive Reprogramming of the Defense Response

The apple genome contains a total of 40,121 genes of which 25,912 were found to be expressed in our data (average expression over all samples > 1 tpm). A total of 10,204 genes (∼39.4% of expressed ones) were differentially expressed on at least one time point studied of which 7,489 exhibited a fold change of at least 2 (∼28.9% of expressed). A transcriptome time series analysis of *Arabidopsis* inoculated with *B. cinerea* found differential expression of about one-third of the *Arabidopsis* genome in the first 48 h (Windram et al., 2012). Our results demonstrate that also in apple extensive reprogramming of the transcriptome occurs upon inoculation with *B. cinerea* and that the extent to which this happened increased in the 28 hpi studied. Furthermore, this started already almost immediately after inoculation and includes some transcriptional activation of secondary metabolism (Figures 1, 2). Almost four times as many genes were significantly upregulated than downregulated. Also in the case of lettuce (*Lactuca sativa*) and strawberry (*Fragaria* × *ananassa*) inoculated with *B. cinerea* a lot more upregulated genes were detected (De Cremer et al., 2013; Xiong et al., 2018).

### Activation of Pathogen-Triggered Immunity in Inoculated Fruit

Our data shows clear evidence that *B. cinerea* inoculated fruit exhibited activation of PTI, which starts with pathogen recognition. In our data, we found upregulation of 12 WAK, 3 CERK1 and one BAK1 genes (Figure 2 and Supplementary Table 5). In lettuce inoculated with *B. cinerea* 12 out of 21 annotated *WAKs* were significantly up or downregulated at 48 hpi (De Cremer et al., 2013). In our data, all differentially expressed *WAKs*, *CERK1s* and *BAK1* were significantly upregulated at 28 hpi and some already at 12 hpi. Brutus et al. (2010) found that *Arabidopsis* overexpressing *WAK1* exhibits enhanced resistance against *B. cinerea*. BAK1 on the other hand plays an important role in PTI signaling and is a negative regulator of cell death (He et al., 2007).

For PTI to manifest, this recognition then needs to be signaled. Evidence that this occurs is provided by the significant overrepresentation in upregulated DEGs of the GO terms protein phosphorylation at 12 and 28 hpi, calcium ion transport at 28 hpi and the KEGG pathway MAPK signaling pathway—plant at 28 hpi (Supplementary Tables 3, 4). More specific evidence is provided by upregulation of 11 *CNGCs*, 5 *CaMs*, 6 *CDPKs*, 1 *CaMK*, 1 *RBOH* and a variety of *MAPKKKs*, *MAPKKs*, and *MAPKs* at 12 and/or 28 hpi (Supplementary Table 5). MEKK1, MKK1/MKK2, and MPK4 form a MAPK cascade that is important in the activation of effective defense against *B. cinerea* in *Arabidopsis* (Qiu et al., 2008). In our data we found two MEKK1, one MKK2 end 3 MPK4 genes that were significantly upregulated. MPK4 may then activate WRKY33 through MKS1, which in turn activates JA/ET responses (Zheng et al., 2006). All four annotated WRKY33 genes in the apple genome were significantly upregulated in our samples. WRKY33 in turn was found to antagonize SA signaling, thus preventing suppression of the JA pathway (Birkenbihl et al., 2012). The WRKY family TFs were the first to show significant overrepresentation around 18 hpi in *Arabidopsis* inoculated with *B. cinerea* (Windram et al., 2012).

Upregulated DEGs at 28 hpi displayed enrichment of regulation of hormone levels and at 12 and 28 hpi of alpha-linoleic acid metabolism (Supplementary Tables 3, 4). This demonstrates that hormonal modulation of fruit defenses did in fact take place and suggests activation of the JA pathway. Closer inspection shows upregulation of JA biosynthesis genes (*LOX, AOS, AOC, OPR3, OPCL1*; Supplementary Figure 7 and Supplementary Table 5). JA works synergistically with ET to coordinate defense responses against necrotrophic pathogens (Memelink, 2009; Bürger and Chory, 2019). Clear activation of ET biosynthesis (*ACS1/2/6, ACO)* and signaling (*ETR, EIN3, ERF1*) could be detected (Supplementary Figure 7 and Supplementary Table 5). Time series analysis of *Arabidopsis* inoculated with *B. cinerea* showed that upregulation of ET biosynthesis through *ACS*, followed by ET signaling, was the first hormonal response at 14 hpi (Windram et al., 2012). We detected significant upregulation of *ACS* at 12 and 28 hpi.

### Metabolic Reprogramming for Defense

#### Non-essential Processes for the Defense Were Downregulated

Downregulation of photosynthesis is a common local response in plant defense as a strategy to free up resources for the defense response and protect photosynthetic machinery from oxidative damage (Bolton, 2009). Our data confirms this with overrepresentation in downregulated DEGs of photosynthesis and biosynthesis of chlorophyll and antenna proteins at 28 hpi. Photosynthesis and biosynthesis of chlorophyll were also downregulated in *Arabidopsis* inoculated with *B. cinerea* starting from 18 to 22 hpi, respectively (Windram et al., 2012).

#### Energy Production Was Transcriptionally Increased to Fuel the Defense

The defense response requires a lot of energy and this can be provided by the upregulation of β-oxidation of fatty acids, glycolysis, pyruvate metabolism, the PDH-bypass, acetyl-CoA biosynthesis, the TCA cycle and the γ-butyric acid (GABA) shunt (Bolton et al., 2008). Key genes of all these pathways were upregulated in our data. We found enrichment of the GO processes lipid metabolic process at 12 and 28 hpi, and acetyl-CoA biosynthetic process and KEGG pathway fatty acid degradation at 28 hpi in upregulated DEGs (Supplementary Tables 3, 4), and upregulation of genes involved in all conversions of the β-oxidation (Supplementary Figure 8 and Supplementary Table 6). Key regulatory points of the glycolysis [*PFK-1*, *hexokinase*, *phosphoglycerate mutase* (*PGM*)] were significantly upregulated (Supplementary Figure 9 and Supplementary Table 6). Our data shows significant upregulation of the transformation of pyruvate into acetyl-CoA by *PDH*, but also through the PDH-bypass that goes through *pyruvate decarboxylase* (*PDC*, 0/6), *aldehyde dehydrogenase* (*ALDH*, 2/10) and *acetyl-CoA synthethase* (2/2). This is confirmed by the overrepresentation of the GO term acetyl-CoA biosynthetic process in upregulated DEGs of 28 hpi (Supplementary Table 3). We suspect activation of the TCA cycle because of significant upregulation of *citrate synthase, aconitate hydratase* and *isocitrate dehydrogenase*. Lastly, upregulation of the GABA shunt is evident from significant upregulation of *glutamate dehydrogenase* (*GDH*) and *glutamate decarboxylase* (*GDC*). In conclusion, we demonstrated that on transcriptional level a clear activation of energy metabolism occurred in *B. cinerea* inoculated apples.

#### Secondary Metabolism Was Activated

Our results show strong activation of terpenoid biosynthesis, which is supported by overrepresentation in upregulated DEGs of isoprenoid metabolic process and terpenoid backbone biosynthesis at 12 hpi, sesquiterpenoid and triterpenoid biosynthesis at 12 and 28 hpi, and monoterpenoid biosynthesis and diterpenoid biosynthesis at 28 hpi (Supplementary Table 3). Terpenoids play a role in plant defense as phytoanticipins and phytoalexins. Our data shows clear activation of the cytosolic MVA pathway (Figure 3 and Supplementary Table 6) which points to biosynthesis of sesqui- and triterpenoids. This is supported by significant upregulation of *FPPS*, *FPPFT*, *SMO*, *(-)-germacrene D synthase*, *β-amyrin synthase* and *β-amyrin 28-monooxygenase*. The MVA pathway was also upregulated in the case of apple-*P. expansum* (Barad et al., 2016), tomato-*Colletotrichum gloeosporioides* (Alkan et al., 2015), lettuce-*B. cinerea* (De Cremer et al., 2013) and strawberry-*B. cinerea* (Xiong et al., 2018). Furthermore, ontogenic resistance in cucumber was linked to terpenoids and terpenoid glycosides in the peel (Mansfeld et al., 2017). *A. alternata* inoculated jujube displayed a red ring phenotype that contained the infection within. One pathway specifically upregulated in this red ring was terpenoid biosynthesis (Yuan et al., 2019). All of this evidence supports a prominent role of terpenoids in the defense response.

A common defense response in fruit is the activation of the phenylpropanoid pathway, e.g., apple-*P. expansum* (Vilanova et al., 2014), strawberry-*B. cinerea* (Xiong et al., 2018) and tomato-*C. gloeosporioides* (Alkan et al., 2015). This is confirmed in our samples by overrepresentation in upregulated DEGs of the GO term phenylpropanoid metabolic process at 0, 12 and 28 hpi, and KEGG pathway phenylpropanoid biosynthesis at 12 and 28 hpi (Supplementary Tables 3, 4). All four homologs of the key enzyme regulating this pathway, PAL, were strongly upregulated in our samples (Figure 4 and Supplementary Table 5). Furthermore, a lot of downstream enzymes in the pathway exhibited significant upregulation.

### Early Activation of the Mevalonate Pathway Correlated With Reduced Susceptibility

Analysis of correlations between gene expression and infection success revealed that the MVA pathway had a strong negative impact on infection success (Figure 5). Especially early activation of this pathway seems to be important. When comparing the progenitor of domesticated apple (*M. sieversii* PI613981) resistant to *P. expansum* with the susceptible domesticated apple cultivar “Royal Gala,” Ballester et al. (2017) found evidence that the early activation of defense responses was one critical factor for resistance. Vilanova et al. (2014) compared the defense of apple in response to a compatible (*P. expansum*) and incompatible (*P. digitatum*) pathogen. Their results showed that in the incompatible interaction, the phenylpropanoid pathway was upregulated faster and stronger. Furthermore, previous research of us has found a link between cell density and resistance in apple, possibly due to a correlation with biosynthetic capacity; meaning, fruit with a higher cell density may be able to mount a larger defense response in a given time (Naets et al., 2019). All this evidence indicates timing and magnitude of defense responses plays a key role in effective defense, and that, in the case of apple-*B. cinerea*, there is an important role for the MVA pathway.

### Ethylene Signaling Affects Secondary Metabolism Needed for Defense

It is generally accepted that resistance against necrotrophs is modulated by JA/ET signaling (Pieterse et al., 2012; Bürger and Chory, 2019). We subjected apples to two treatments that can impact the ET component. 1-MCP is an ET perception inhibitor that strongly binds the ET receptor without activating it. Heat treatments can inactivate *ACS* and *ACO* which are heat labile, and even impact ET signaling by either inactivation of the receptors or affecting the downstream signaling (Lurie, 1998). For 1-MCP, this is confirmed by a significant reduction in *ERF1* expression in peel at 12 and 29 w and in flesh at 12 w. HWD5 did not significantly affect *ERF1* expression, but did significantly reduce *MPK3* and *MPK6* expression in peel tissue at harvest. *MPK3* and *MPK6* play a role in *Botrytis*-induced ET biosynthesis, but also in downstream activation of ET responsive genes (Jagodzik et al., 2018). *PAL1*, *SMO1*, and *SMO2* were tested as key genes for the phenylpropanoid and terpenoid biosynthesis. Both 1-MCP and HWD5 significantly affected expression of *PAL1* and *SMO1*, demonstrating that ET likely plays a role in regulating both pathways.

Both 1-MCP and HWD5 significantly increased lesion diameters 96 hpi and this for all tested time points throughout CA storage. Resistance against *B. cinerea* in *Arabidopsis* has been linked to the ability to confine the infection by cell wall modifications using hydroxycinnamates and monolignols. This defense response was mediated by ET (Lloyd et al., 2011). Therefore, increased susceptibility in 1-MCP treated fruit could be a result of a lessened ability to confine the pathogen due to the significant reduction in *ERF1* and *PAL1* expression. *PAL1* was also significantly lowered in peel tissue of HWD5 treated fruit at harvest, but not at 29 w. It is possible that this resulted in a lower amount of PAL1 proteins, even after expression levels recovered. Both 1-MCP and HWD5 resulted in significantly lower expression of *SMO1* in peel tissue at each time point. The log_2_ reduction in peel tissue of 1-MCP treated fruit was -0.8 ± 0.29 and -1.61 ± 0.25 at 12 and 29 w, respectively, and in peel tissue of HWD5 treated fruit was -0.43 ± 0.15 and -0.62 ± 0.19 at harvest and 29 w, respectively. This difference in effect size could then also explain the difference in susceptibility increase. In practice *B. cinerea* can cause latent infections of flowers that then develop later during storage and fruit ripening (Dean et al., 2012), is able to directly infect fruit in the orchard *via* natural openings, operates as wound pathogen taking opportunity of wounds caused by insects, birds or postharvest handling (Droby and Lichter, 2007; Carisse, 2016), and can spread from fruit to fruit in the storage room in what is called nest rot (Snowdon, 1990). It can be expected that these different infection pathways, fruit maturity at infection, and disease development conditions may have their impact on these results.

## Conclusion

In this work we presented an RNAseq study of *B. cinerea* inoculated apple. We demonstrate that drastic reprogramming occurs upon inoculation with differential expression of 28.9% of expressed genes. Part of this was a complete transcriptional reprogramming of the metabolism with a downregulation of photosynthesis and an upregulation of energy and secondary metabolism. Here we provided evidence of a correlation between an early activation of the mevalonate pathway that synthesizes precursors for terpenoids and reduced susceptibility. Further evidence for the importance of terpenoids in the defense response is provided by treating fruit with 1-MCP or HWD5. These treatments impacted ET perception and signaling, potentially related with the observed significantly larger lesions at 96 hpi. We demonstrated a significant reduction for both treatments in *SMO1* expression in peel tissue, a key gene in terpenoid biosynthesis. This evidence points to an important impact of terpenoid biosynthesis coordinated by ET signaling on apple defense against *B. cinerea*.

## Data Availability Statement

The data presented in the study are deposited in the GEO repository, accession number GSE195841.

## Author Contributions

MN: substantial contribution to conception and design, execution, analysis, and interpretation. WV: substantial contribution to acquisition of the data of the work. WG, PV, and BN: substantial contribution to analysis of the data. WK and AG: substantial contribution to design and interpretation. BD: substantial contribution to interpretation. All authors contributed to the article and approved the submitted version.

## Conflict of Interest

The authors declare that the research was conducted in the absence of any commercial or financial relationships that could be construed as a potential conflict of interest.

## Publisher’s Note

All claims expressed in this article are solely those of the authors and do not necessarily represent those of their affiliated organizations, or those of the publisher, the editors and the reviewers. Any product that may be evaluated in this article, or claim that may be made by its manufacturer, is not guaranteed or endorsed by the publisher.

## Footnotes

1
www.rosaceae.org
