# Supplementary material for: Time Is of the Essence—Early Activation of the Mevalonate Pathway in Apple Challenged With Gray Mold Correlates With Reduced Susceptibility During Postharvest Storage
Source: Front Microbiol. 2022 May 12;13:797234. doi: 10.3389/fmicb.2022.797234 (PMC9133740; doi:10.3389/fmicb.2022.797234)
Supplement: Supplementary file 11 [file Table_11.docx]

**Supplementary Figure 1:** Example of an inoculated and sampled ‘Jonagored’ apple from the RNAseq experiment.

**Supplementary Figure 2:** Scatter plots of replicate ‘Jonagored’ samples of 0 hpi. Each point represents the regularized log transformed count of a gene for both samples. C: Control; M: Mock inoculated; I: Botrytis inoculated; 00: 0 hpi; A1-3: Replicate 1-3.

**Supplementary Figure 3:** Scatter plots of replicate ‘Jonagored’ samples of 1 hpi. Each point represents the regularized log transformed count of a gene for both samples. C: Control; M: Mock inoculated; I: Botrytis inoculated; 01: 1 hpi; A1-3: Replicate 1-3.

**Supplementary Figure 4:** Scatter plots of replicate ‘Jonagored’ samples of 12 hpi. Each point represents the regularized log transformed count of a gene for both samples. C: Control; M: Mock inoculated; I: Botrytis inoculated; 12: 12 hpi; A1-3: Replicate 1-3.

**Supplementary Figure 5:** Scatter plots of replicate ‘Jonagored’ samples of 28 hpi. Each point represents the regularized log transformed count of a gene for both samples. C: Control; M: Mock inoculated; I: Botrytis inoculated; 28: 28 hpi; A1-3: Replicate 1-3.

**Supplementary Figure 6:** Significantly enriched (a) GO terms of biological processes and (b) KEGG pathways in ‘Jonagored’ apples inoculated with *B. cinerea*. The test set consists of up regulated DEGs determined by DESeq2 and the reference set consists of all genes with an average expression of at least 1 tpm in all control and mock samples.

**Supplementary Figure 7:** Overview of the regulation of hormone signaling pathways in ‘Jonagored’ apple inoculated with *B. cinerea*, and their interactions. ABA: Abscisic acid; NCED1: 9-Cis-epoxycarotenoid dioxygenase 1; AAO3: Abscisic aldehyde oxidase; ABA8H: ABA 8’-hydroxylase; PYR/PYL: ABA receptor PYR/PYL family; PP2C: Protein phosphatase 2C; SnRK2: Serine/threonineprotein kinase SRK2; ABF: ABA responsive element binding factor; MYC: Transcription factor MYC; JA: Jasmonic acid; LOX: Lipoxygenase; AOS: Allene oxide synthase; AOC: Allene oxide cyclase; OPR3: 12-Oxo-cis-10,15-phytodienoic acid reductase; OPCL1: 3-Oxo-2-(cis-2’-pentenyl)-cyclopentane-1-octanoic acid-8:0 CoA ligase; JAAH: Jasmonoyl-L-amino acid hydrolase; COI1: Coronatine insensitive 1; SCFCOI1: Skp, Cullin, F-box containing complex bound with COI1; JAZ: Jasmonate zim domain protein; ET: Ethylene; ACS1/2/6: 1-Aminocyclopropane-1-carboxylate synthase 1/2/6; ACO: 1-Aminocyclopropane-1-carboxylate oxidase; ETR: ET receptor; EIN3: ETinsensitive protein 3; ERF1: ET-responsive transcription factor 1; PAL: Phenylalanine ammonia-lyase; SA: Salicylic acid; NPR1: Nonexpresser of PR genes 1; TGA: TGACG binding factor 1; MPK4: Mitogen-activated protein kinase 4; 1-MCP: 1-Methylcyclopropene.

**Supplementary Figure 8:** Overview of the regulation of the β-oxidation of fatty acids in *B. cinerea* inoculated ‘Jonagored’ apples. Heat maps were constructed from genes with significant differential expression. The log2 fold change is relative to mock inoculation. ADH: Alcohol dehydrogenase; ALDH: Aldehyde dehydrogenase; TAG: Triacylglycerol.

**Supplementary Figure 9:** Overview of the regulation of the glycolysis in *B. cinerea* inoculated ‘Jonagored’ apples. Heat maps were constructed from genes with significant differential expression. The log2 fold change is relative to mock inoculation. G6P1E: Glucose-6P isomerase 1; PFK-1: Phosphofructokinase-1; FBP: Fructose 1,6-bisphosphate; GA3P: Glyceraldehyde 3-phosphate; DHAP: Dihydroxyacetone phosphate; GAPDH: GA3P dehydrogenase; 1,3BPG: 1,3-Bisphosphoglycerate; 3PG: 3-Phosphoglycerate; PGM: Phosphoglycerate mutase; 2PG: 2-Phosphoglycerate; PEP: Phosphoenolpyruvate; PEPC: PEP carboxylase; LDH: Lactate dehydrogenase; DAHP: 3-Deoxy-arabino-heptulonate 7-phosphate; PDH: Pyruvate dehydrogenase.

**Supplementary Figure 10:** Correlation of lesion diameter of the middle spot with the average of the outer two spots in *B. cinerea* inoculated ‘Jonagored’ apples. The blue line represents the 1:1 line and the red line the linear fit through the data (adjusted R^2^ = 0.78, pearson correlation = 0.884, p < 0.001). The gray area around the red line represents the standard error of the linear fit.
