# Supplementary material for: Time Is of the Essence—Early Activation of the Mevalonate Pathway in Apple Challenged With Gray Mold Correlates With Reduced Susceptibility During Postharvest Storage
Source: Front Microbiol. 2022 May 12;13:797234. doi: 10.3389/fmicb.2022.797234 (PMC9133740; doi:10.3389/fmicb.2022.797234)
Supplement: Supplementary file 12 [file Table_12.docx]

**Supplementary Table 1:** Primer sequences for RT-qPCR targets.

**Supplementary Table 2:** Sequencing information of all the samples.

**Supplementary Table 3:** GO enrichment analysis.

**Supplementary Table 4:** KEGG enrichment analysis.

**Supplementary Table 5:** Overview of genes involved in PTI. Whenever a gene exhibited significant differential expression, the LFC is displayed.

**Supplementary Table 6:** Overview of genes involved in primary and secondary metabolism. Whenever a gene exhibited significant differential expression, the LFC is displayed.

**Supplementary Table 7:** Overview of DESeq2 analysis results for all genes. For each gene, the LFC, *p*-value (pval), false discovery rate (FDR) is displayed for each time point studied for either the comparison of *Botrytis* inoculated with the control (I vs C) or with the mock (I vs M). If a gene was found to be significant, this was indicated with a ‘1’ and the direction of regulation (up or down) is then also displayed.

**Supplementary Table 8:** Relative abundance of compounds by GC-MS analysis. Identifications was done using NIST 17 Mass Spectral Library, but are not absolute.

**Supplementary Table 9:** Relative abundance of compounds by LC-MS analysis.

**Supplementary Table 10:** ANOVA results of RT-qPCR data.
