# Supplementary figures and images for: Time Is of the Essence—Early Activation of the Mevalonate Pathway in Apple Challenged With Gray Mold Correlates With Reduced Susceptibility During Postharvest Storage
Source: Front Microbiol. 2022 May 12;13:797234. doi: 10.3389/fmicb.2022.797234 (PMC9133740; doi:10.3389/fmicb.2022.797234)

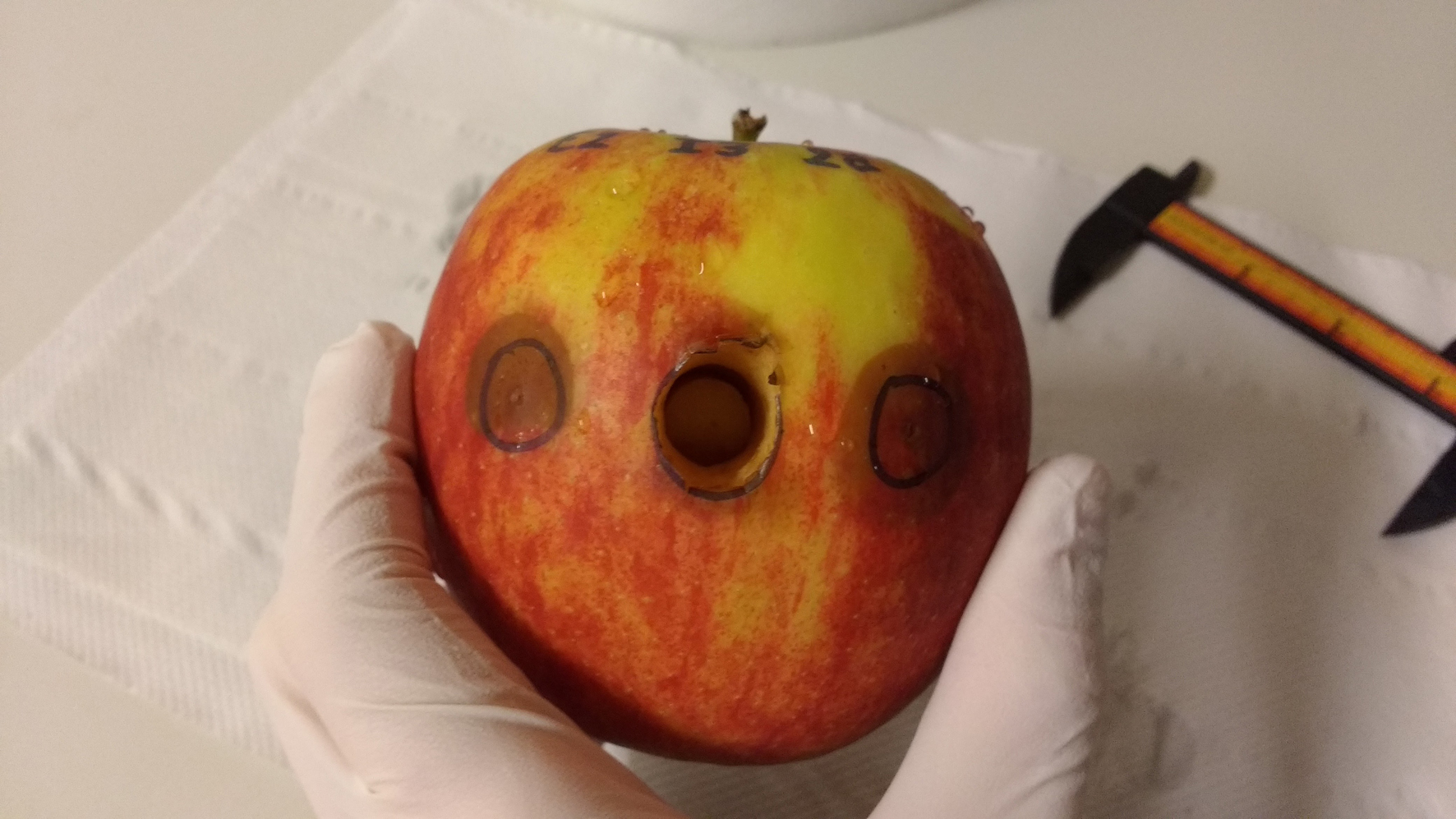

Supplement: Supplementary file 13 [file Image_1.JPEG]

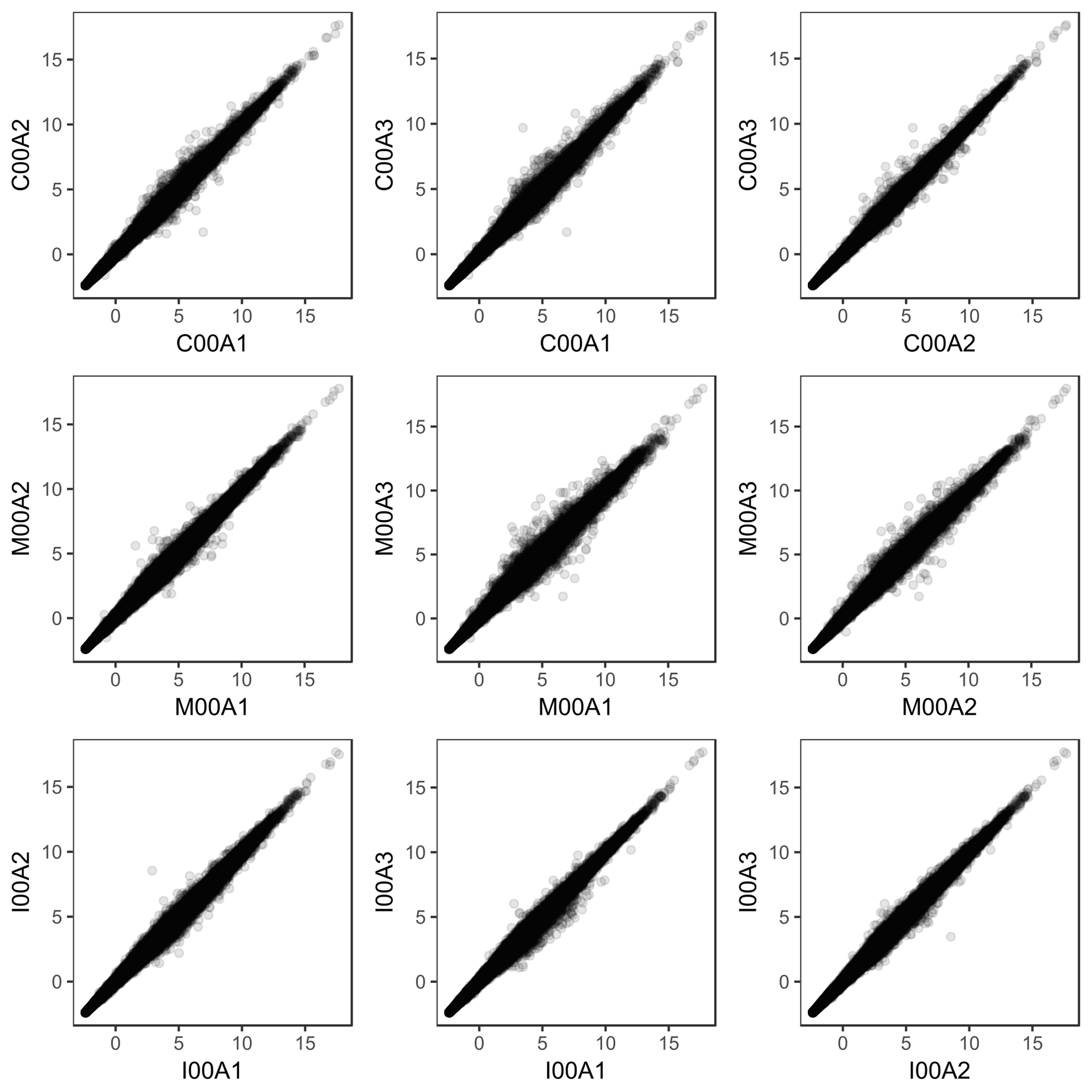

Supplement: Supplementary file 14 [file Image_2.PNG]

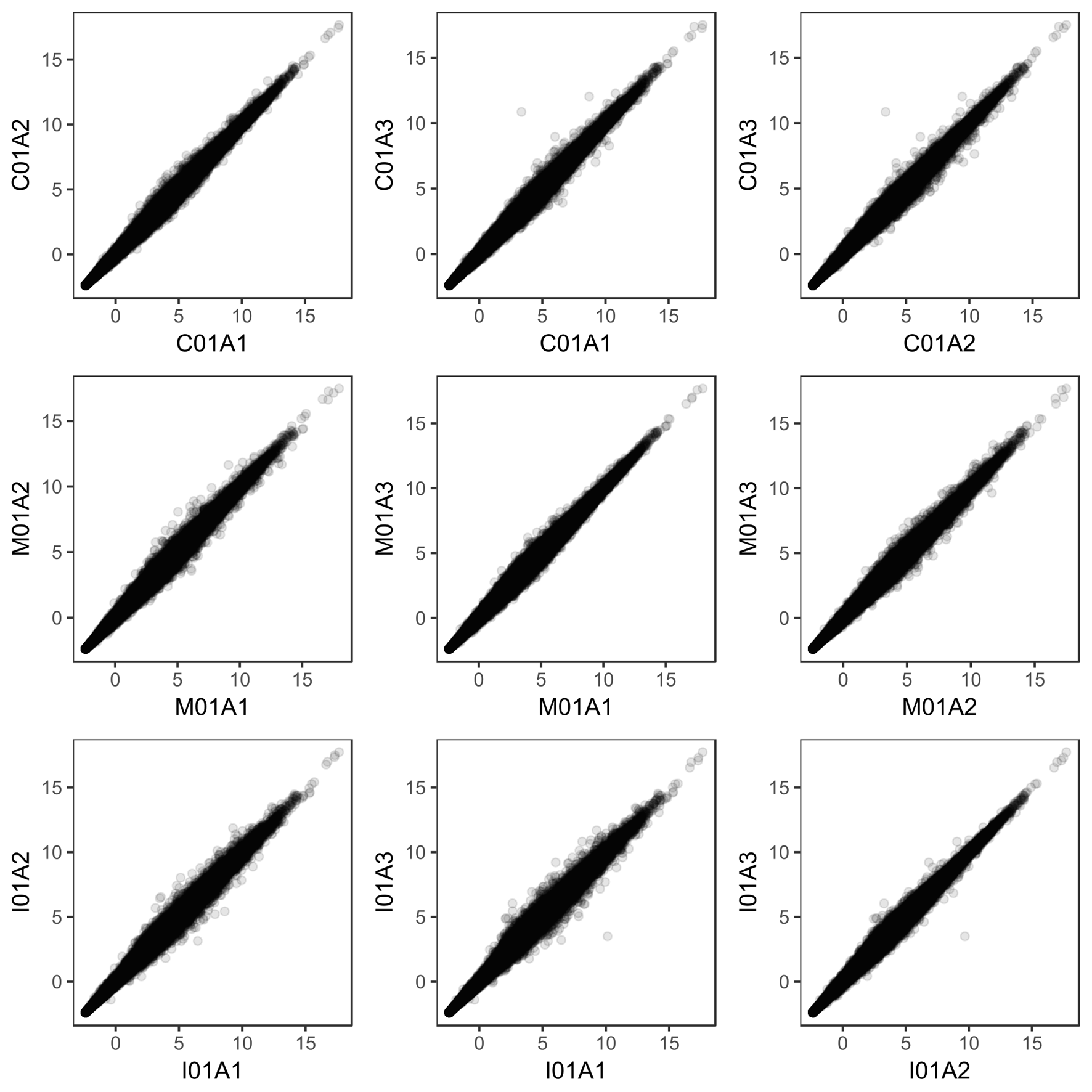

Supplement: Supplementary file 15 [file Image_3.PNG]

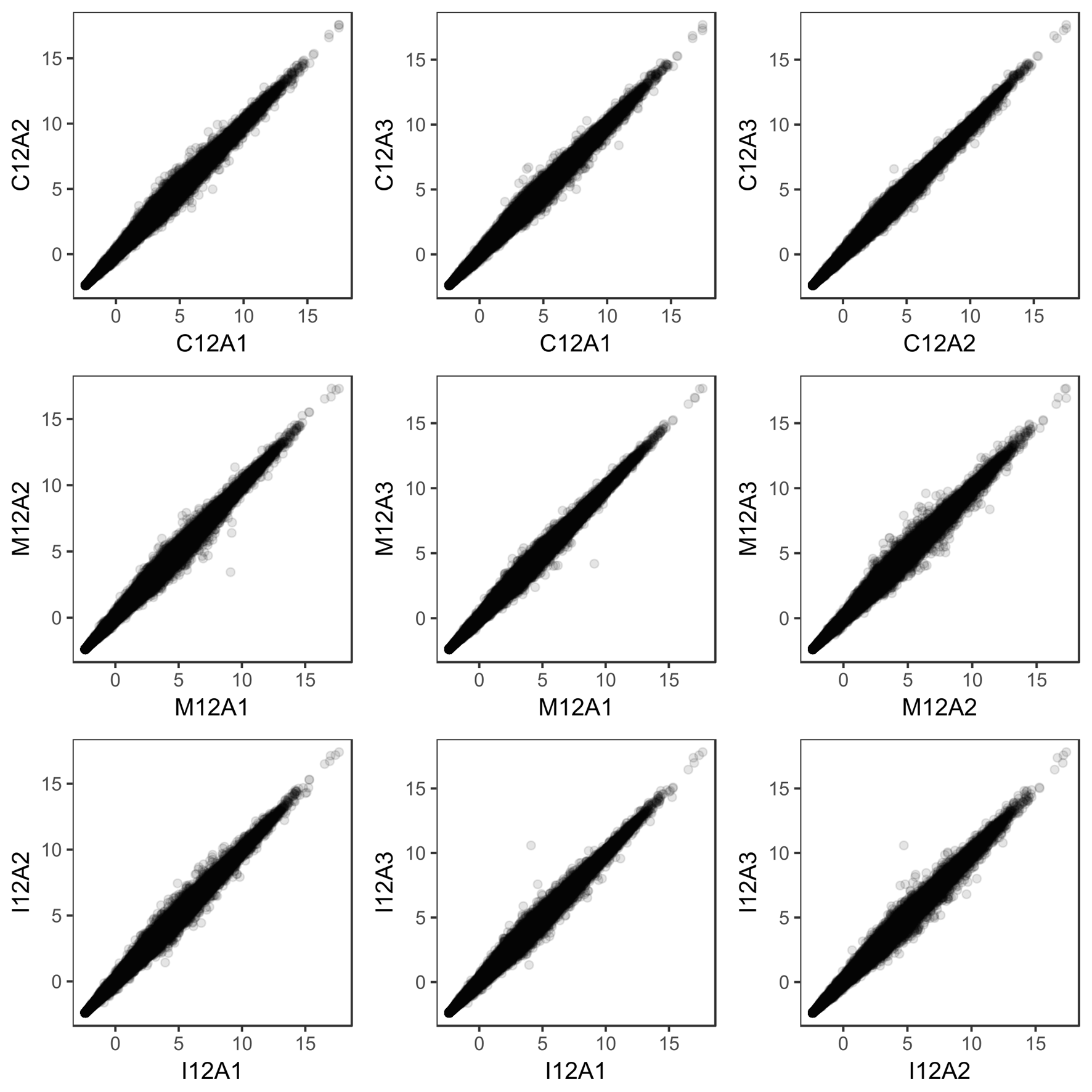

Supplement: Supplementary file 16 [file Image_4.PNG]

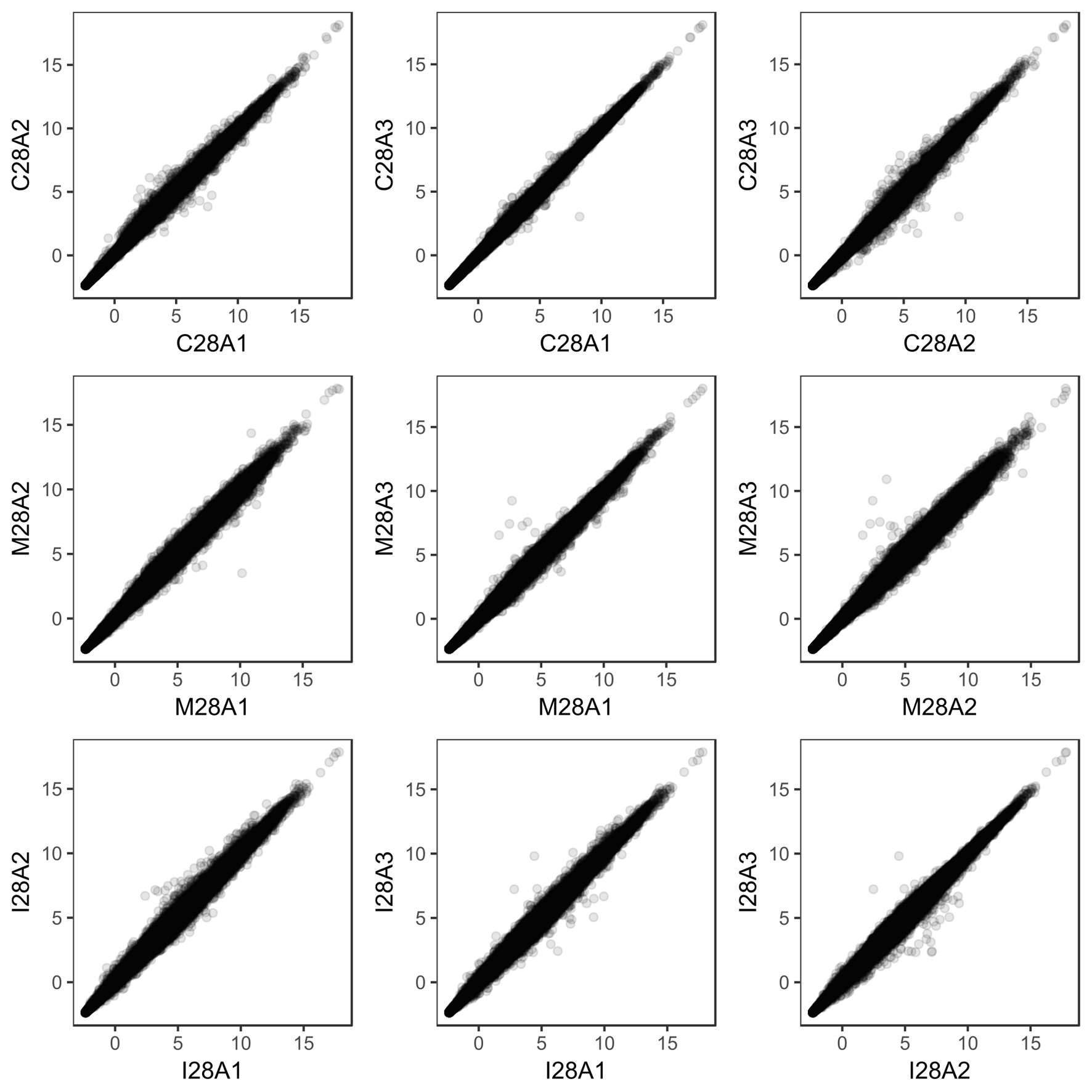

Supplement: Supplementary file 17 [file Image_5.PNG]

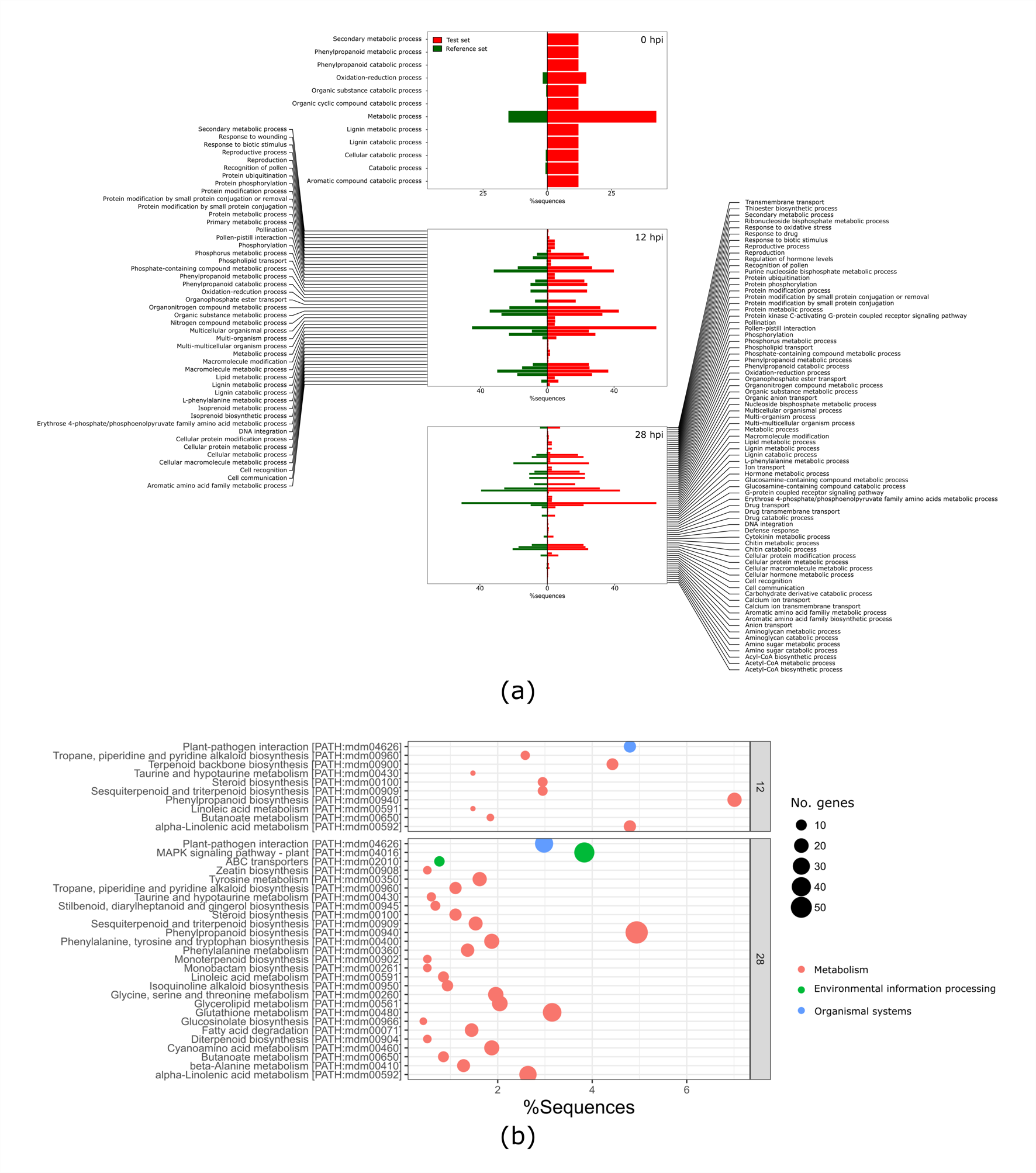

Supplement: Supplementary file 18 [file Image_6.PNG]

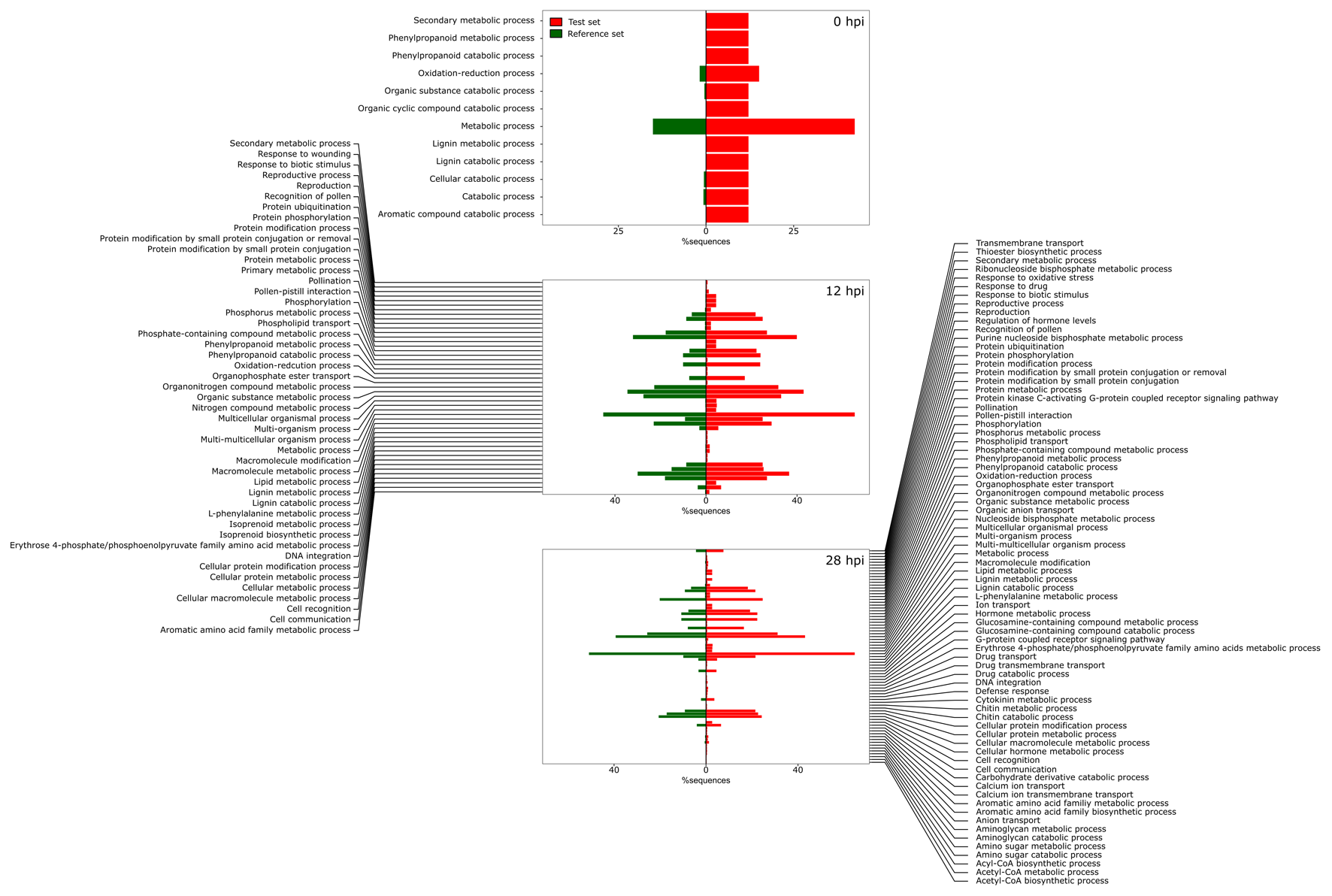

Supplement: Supplementary file 19 [file Image_7.PNG]

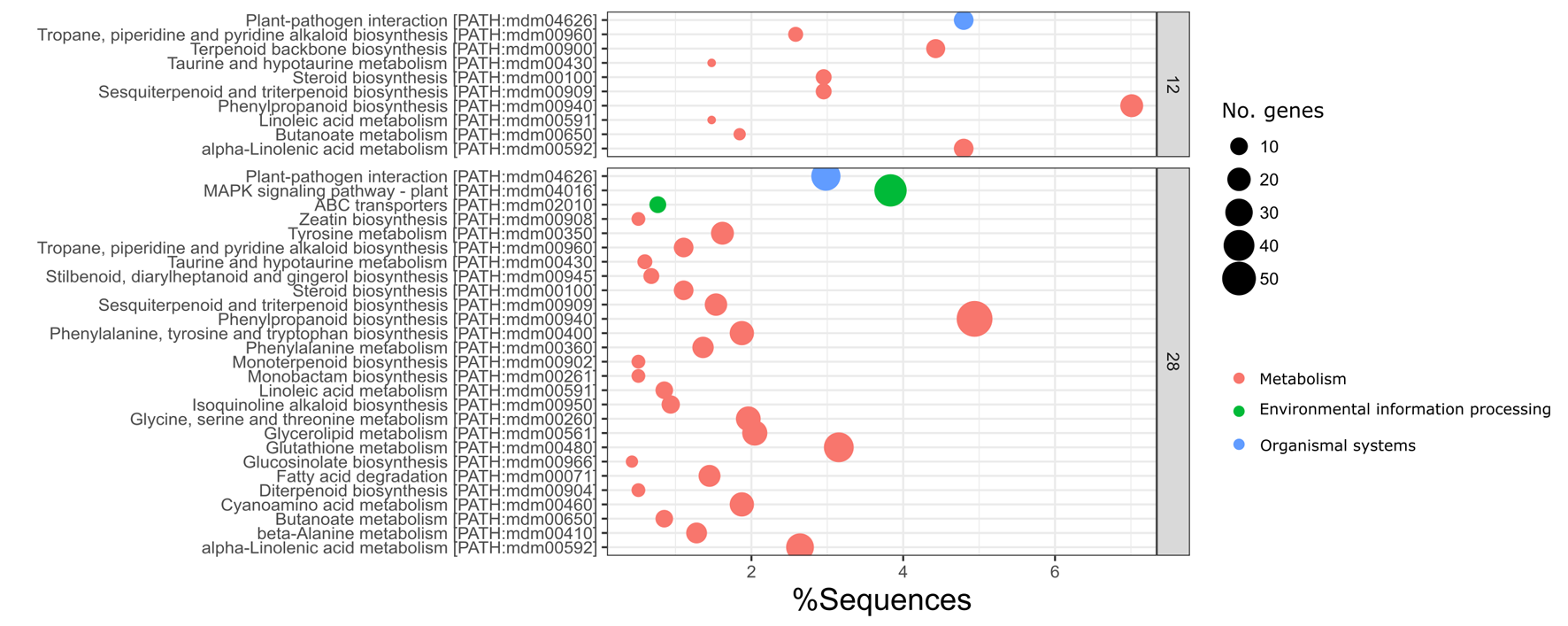

Supplement: Supplementary file 20 [file Image_8.PNG]

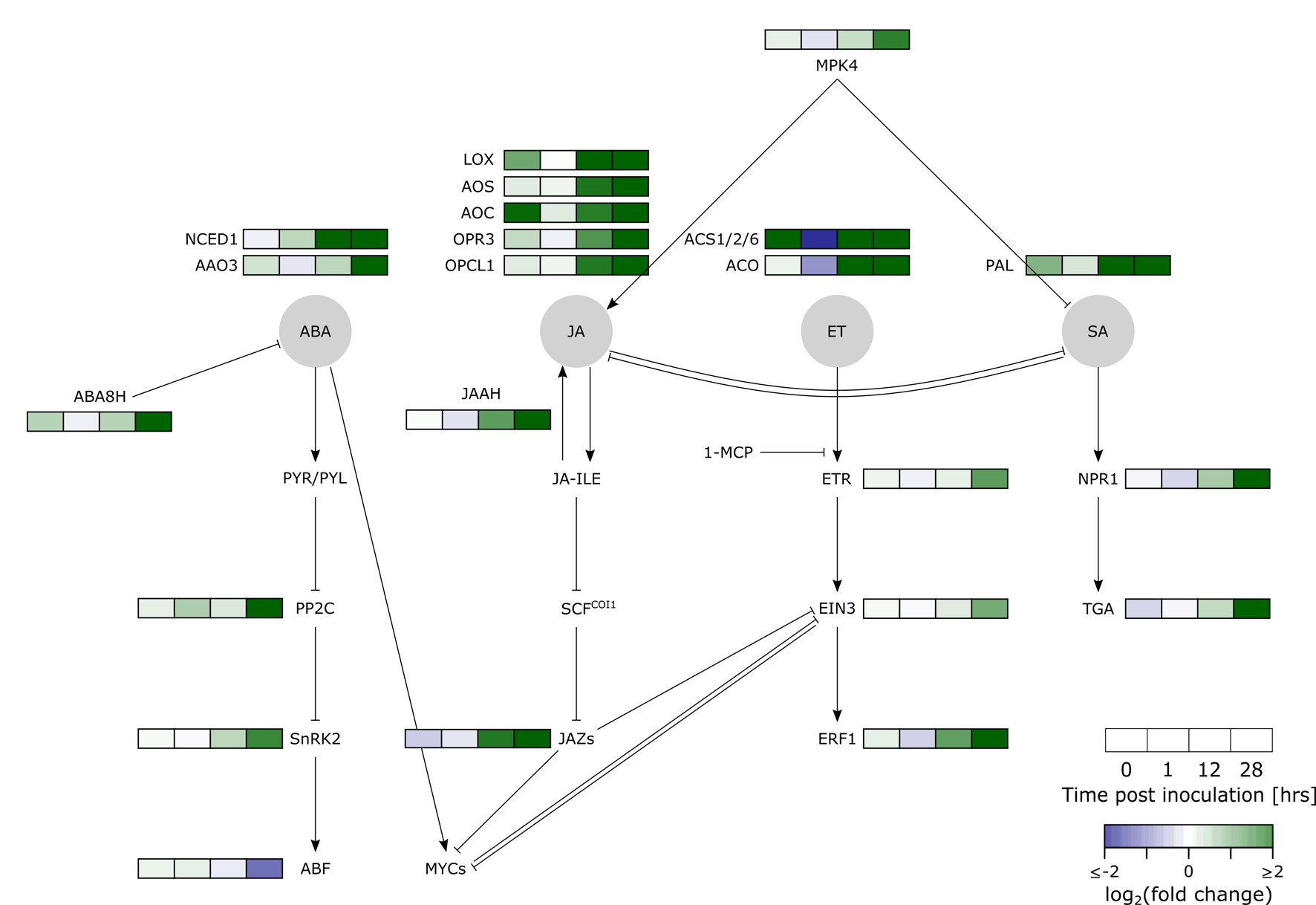

Supplement: Supplementary file 21 [file Image_9.PNG]

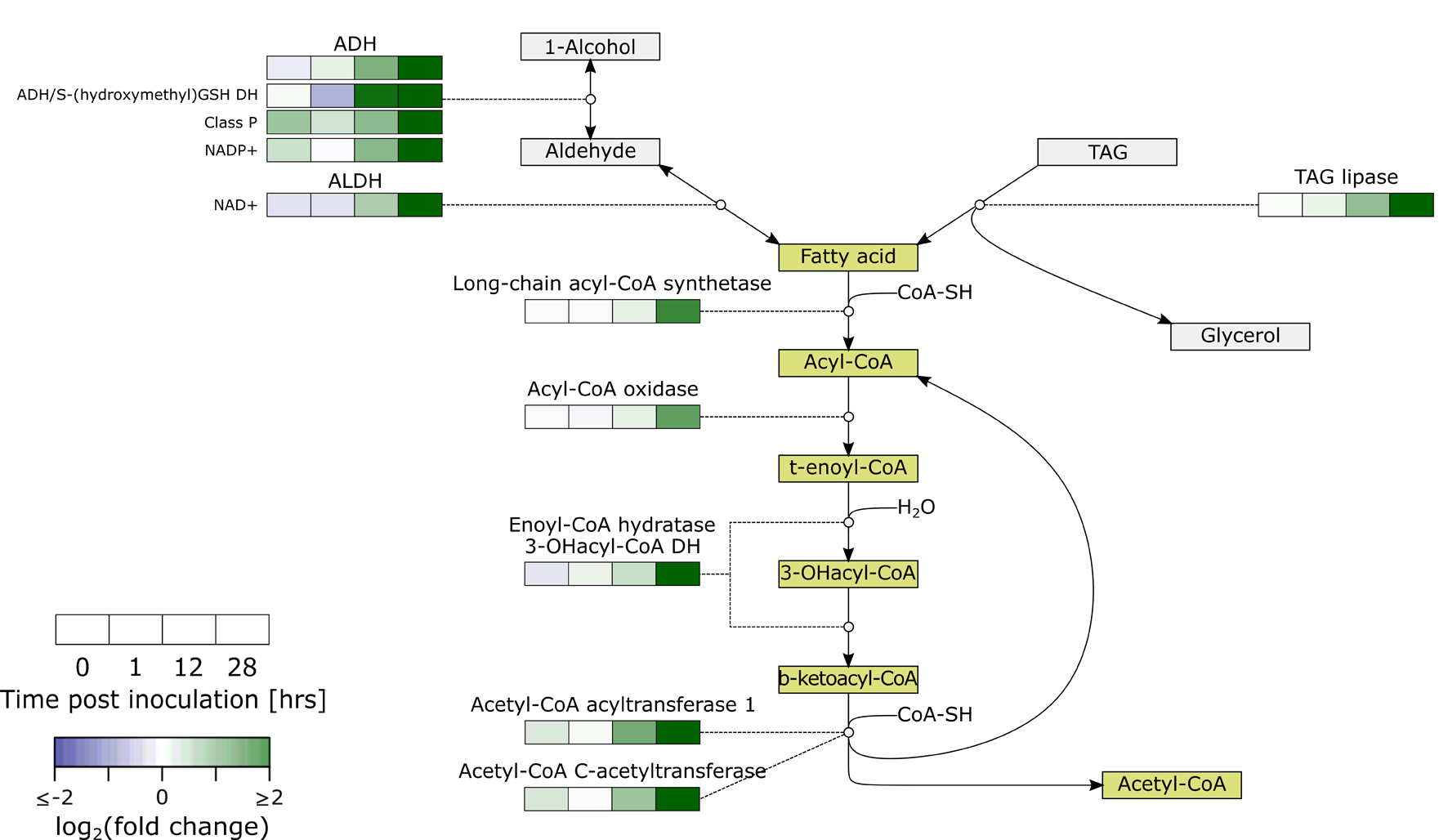

Supplement: Supplementary file 22 [file Image_10.PNG]

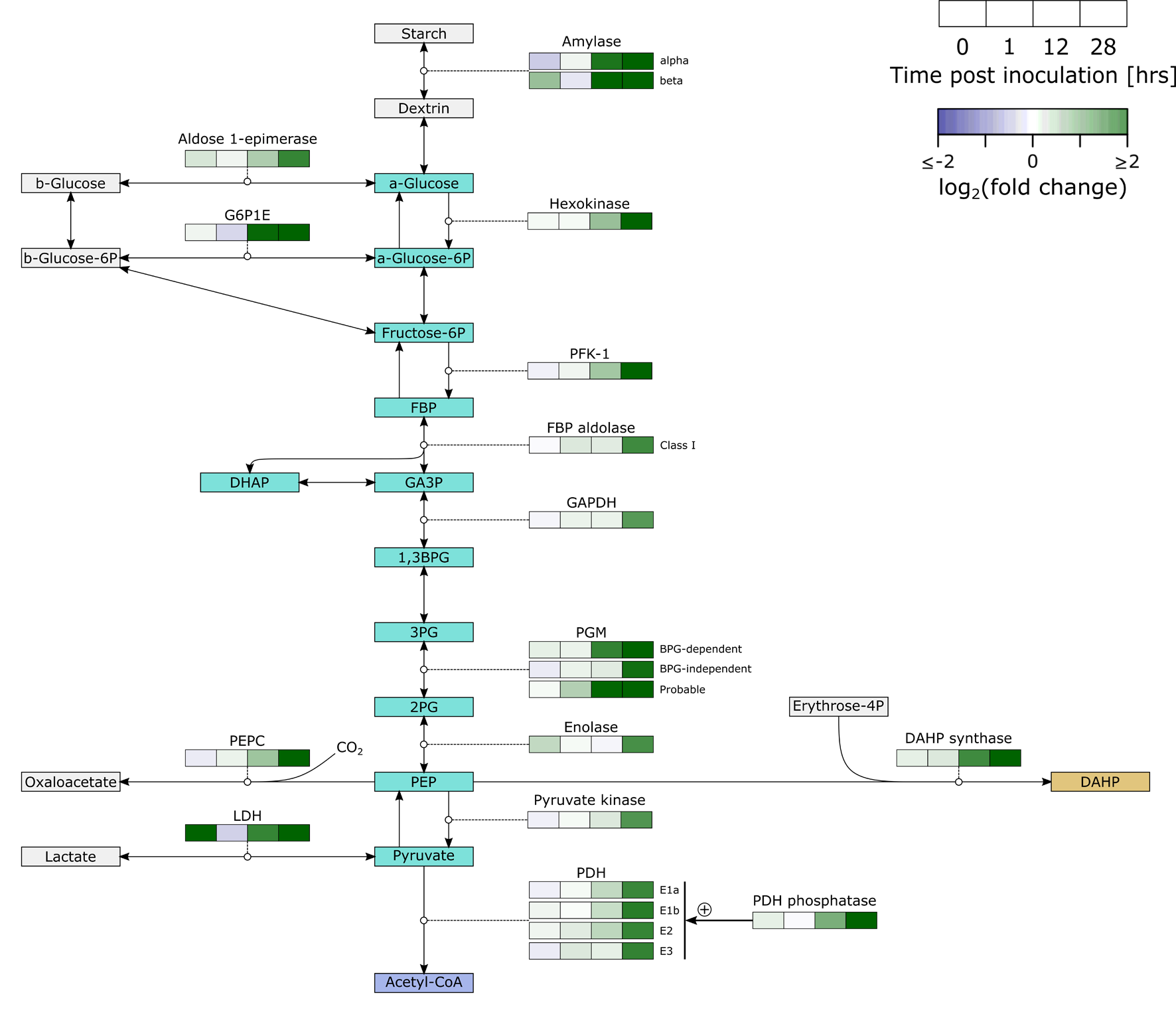

Supplement: Supplementary file 23 [file Image_11.PNG]

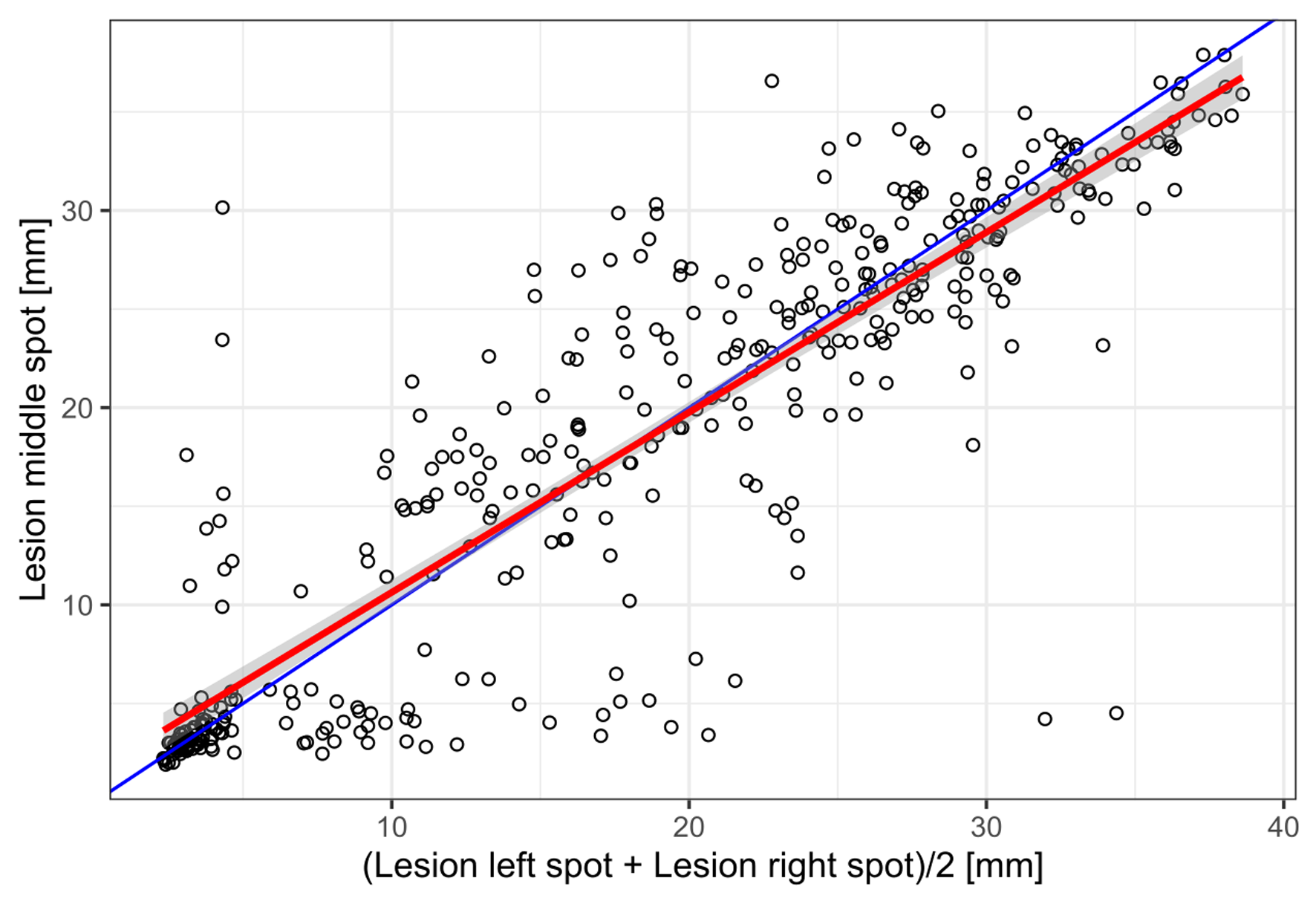

Supplement: Supplementary file 24 [file Image_12.PNG]
